# Bending‐Resistant Intimate 3D Graphene–Metal Heterojunctions for Highly Sensitive and Robust Flexible Sensors

**DOI:** 10.1002/advs.202600080

**Published:** 2026-07-02

**Authors:** Saeyoung Park, Yoo‐Kyum Shin, Na‐Kyoung Yang, Gyeong‐Hwan Park, Somi Lee, Min‐Ho Seo

**Affiliations:** ^1^ Department of Information Convergence Engineering College of Information and Biomedical Engineering Pusan National University Yangsan Republic of Korea; ^2^ School of Biomedical Convergence Engineering College of Information and Biomedical Engineering Pusan National University Yangsan Republic of Korea

**Keywords:** electrochemical biosensor, flexible electronics, heterojunction, interconnection, LIG, wearable sensor

## Abstract

Three‐dimensional (3D) graphene offers exceptional electrical and mechanical properties at the material level, yet these advantages are often compromised during system integration due to the lack of a reliable, miniaturizable interfacial method with conventional electronics. In particular, mismatched interfacial properties and the absence of robust interconnection techniques have hindered seamless implementation at the system level, stalling progress toward miniaturization and practical applications. Here, we present a localized interconnection method in which conventional silver nanoparticles (Ag‐NPs) ink is deposited into a reservoir‐structured metal electrode under a controlled thermal environment, selectively accelerating solvent evaporation to yield high electrical conductivity and mechanical robustness. This approach enables the formation of micrometer‐scale interconnections with minimal spreading, while achieving low contact resistance (7.14 Ω), stable impedance (< 10^5^ Hz), and high mechanical durability under repeated bending at a 2 mm radius of curvature, along with excellent environmental stability. Finally, we applied the proposed method to high‐performance wearable multi‐modal motion sensors and electrochemical biosensors, demonstrating its utility in emerging applications, such as human‐robot‐interaction and point‐of‐care diagnostics.

## Graphical abstract

A reservoir‐guided silver‐nanoparticle soldering strategy is developed for bending‐resistant 3D graphene–metal heterojunctions. Controlled hot‐plate evaporation confines conductive ink within the metal reservoir, suppressing spreading and protecting the LIG interface from deformation‐induced fracture. This intimate interconnection enables robust, flexible devices, including wearable bending sensors and miniaturized electrochemical biosensors.

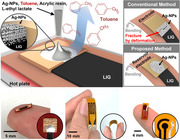

## 1 Introduction

Recent advances in electronics have focused on achieving higher performance, miniaturization, and mechanical flexibility, thereby significantly broadening their range of applications. In particular, miniaturized and flexible electronic devices enable unprecedented functionalities previously unattainable with conventional rigid‐substrate electronics, owing to their ability to conform to various surfaces and operate without structural constraints. These advantages have been practically demonstrated across a wide range of fields, including robotics [1, 2], automotive and aerospace systems [3], electronic skin [4], biomedical devices [5, 6], environmental monitoring [7], wearable healthcare [8, 9, 10], and next‐generation displays [11], positioning flexible electronics as a key technology in the development of future electronic systems.

In this context, carbon or graphene‐based materials have emerged as key building blocks for flexible electronics and sensors, as they can support both mechanical sensing [12, 13, 14, 15] (e.g., strain and pressure) and electrochemical signal transduction for biochemical analytes such as metabolites and biomarkers [16]. Various graphene fabrication methods have been extensively explored, including mechanical exfoliation [17], chemical vapor deposition (CVD) [18], liquid‐phase exfoliation (LPE) [19], and reduction of graphene oxide (rGO) [20]. However, these approaches often involve high‐temperature or vacuum systems, metal catalysts, multiple transfer steps, or produce graphene with substantial defects that limit their direct integration with flexible devices.

In contrast, the laser‐induced process enables direct, one‐step graphene formation on diverse flexible substrates under ambient conditions, without the need for vacuum environments, catalysts, or post‐transfer procedures [21]. This method offers rapid, mask‐free micropatterning and high manufacturing freedom, while the intrinsic three‐dimensional porous morphology of LIG provides excellent electrical, mechanical, and chemical properties ideal for miniaturized and high‐performance flexible sensor applications.

However, despite these advancements, the reliability of carbon–metal interconnections remains a critical bottleneck for practical implementation. Hydrogel‐based conductors and hybrid material systems improve mechanical adaptability, self‐healing, and long‐term wearable stability, as demonstrated in self‐healing and adhesive ionic hydrogels [22], in‐ear adaptive electronics [23], and cardiovascular wearable sensors [24], while thermally compatible epidermal hydrogel sensors [25] ensure prolonged device operation. In addition, multimodal photonic or strain‐engineered systems enhance sensing capability [26, 27, 28], and flexible and stretchable architectures, including next‐generation wearable platforms such as biodegradable systems, humidity‐insensitive e‐noses, and intelligent health‐monitoring devices [29, 30, 31], illustrate the expanding landscape of miniaturized and mechanically robust electronics. Yet the development of stable and miniaturizable electrical contacts between porous carbon structures and metal conductors has received relatively little attention. Under cyclic mechanical deformation, interfacial stress concentration between dissimilar materials induces crack propagation and electrical degradation, ultimately limiting device lifespan and scalability [32, 33, 34, 35]. As device dimensions shrink and interconnect density increases, these failure mechanisms become increasingly severe. This challenge becomes particularly critical in emerging miniaturized and flexible devices, where reliable interconnections are essential for stable operation. In particular, LIG–metal interfaces face similar demands, as maintaining both electrical and mechanical integrity is vital for high‐performance, wearable, and implantable systems.

To address these challenges, several approaches have been proposed to interconnect LIG with metal electrodes, including gold (Au) deposition [36, 37], copper (Cu) tape attachment [38], conductive paste/ink application [39, 40], and their combinations [41, 42, 43]. However, these existing methods still suffer from limitations in reproducibility, durability, and cost‐effectiveness. In particular, most are manually applied, resulting in poor electrical contact quality and insufficient mechanical robustness. Conventional conductive inks, while capable of forming good electrical interfaces, require the use of additional LIG traces that may act as parasitic impedances. Furthermore, the ink's tendency to spread uncontrollably increases the interconnection area, which is detrimental to miniaturization. Therefore, the development of advanced interconnection technologies that are both electrically and mechanically stable is essential for the practical deployment and industrialization of LIG‐based ultra‐compact and high‐performance electronic devices.

Herein, we present a method for forming an electrically conductive and mechanically robust, micrometer‐scale miniaturized interconnection between LIG and metal electrodes. This is achieved by precisely dispensing a low‐viscosity silver nanoparticle (Ag‐NPs) ink into the interior of a micromachined reservoir‐structured 3D metal electrode. The reservoir physically confines the ink, effectively suppressing excessive spreading and enabling spatially controlled “selective evaporation” of the solvent. Specifically, the developed selective evaporation process leverages the temperature‐vapor pressure relationship of the solvent to precisely regulate the processing temperature, thereby achieving uniform solidification of Ag‐NPs and micrometer‐scale soldering with high reproducibility. The resulting interconnection exhibits ideal electrical and mechanical contact characteristics, achieving contact resistance below a few ohms over a localized area, and enabling stable transmission of low‐amplitude electrical signals without loss. Additionally, the reservoir structure mechanically reinforces the interconnection, significantly improving its durability under repeated mechanical deformation. Finite Element Method (FEM) simulations and cyclic bending tests confirm that the structure effectively distributes stress, enhancing the structural reliability of the device on flexible substrates. To validate the practical utility of the proposed method, we implemented two miniaturized LIG‐based application devices. The first is a wearable bending sensor for robot control, which demonstrated stable and precise motion signal output under various deformation conditions, enabling reliable human–robot interaction. The second is a mobile electrochemical biosensor for uric acid (UA) detection, which showed excellent sensitivity, selectivity, and reproducibility, highlighting the method's potential for portable biomedical applications.

## 2 Results and Discussion

**FIGURE 1:**
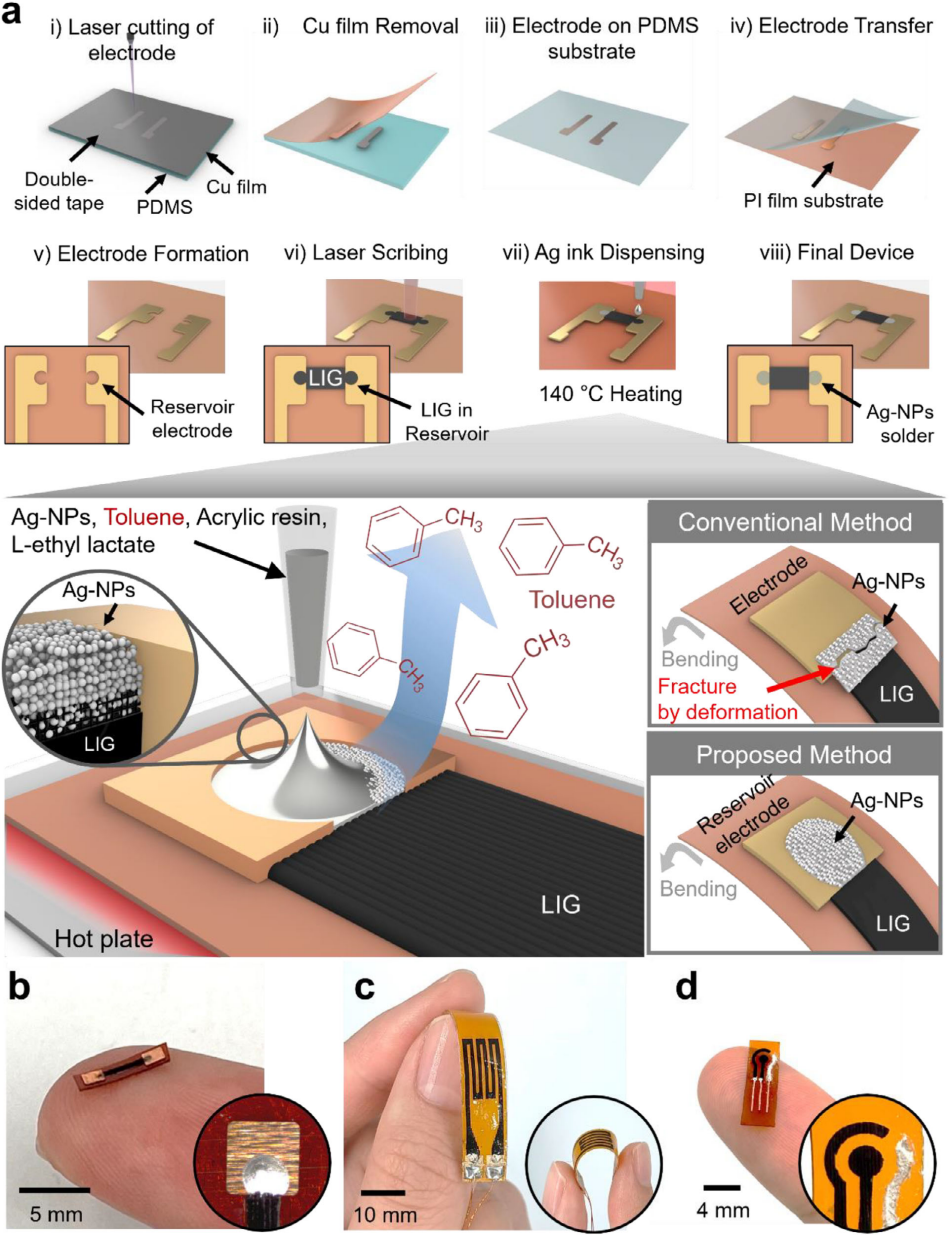
A stable LIG interconnection Method for various applications. (a) Schematic illustration of the proposed fabrication process. (b) Miniaturized resistor with the LIG interconnection (inset: magnified image of the interconnection). (c) Fabricated flexible LIG bending sensor. (d) Fabricated miniaturized LIG‐based three‐electrode electrochemical sensor.

Figure 1a is a schematic diagram of the proposed interconnection method. The method begins with forming copper film electrodes on a flexible polyimide (PI) film substrate (Figure 1ai‐v). These thick Cu electrodes were patterned by UV laser cutting and fabricated via a transfer method, featuring a concave “reservoir” geometry. The electrode thickness and surface roughness were also measured, and the results are provided in Figure S1. The formed Cu electrode is characterized by several tens of micrometers in thickness and a concave reservoir shape that extends inward. After forming this reservoir structure electrode on the substrate, when a CO_2_ laser irradiates the PI substrate area, including the inner area of the metal reservoir, the laser undergoes photothermal and photochemical reactions with the PI substrate along the path to generate 3D graphene. Although the laser passes through the PI substrate and the Cu electrode, LIG continuously formed only on the exposed PI area without being physically or chemically damaged on the Cu electrode because Cu exhibits negligible absorption of CO_2_ laser radiation (Figures 1a‐ii) (Figure S2). Moreover, when LIG is formed on the PI substrate adjacent to the electrode, high temperatures of several hundred degrees or more may occur momentarily, but the large heat capacity due to the sufficient thickness of the Cu electrode can prevent damage to the electrode (Figure S3).

In the next step, Ag‐NPs ink (ELCOAT Silver Resin Paste, C‐510, CANS, Japan) is applied inside the reservoir using a micropipette and then soldered to form a practical electrical bond, which refers to the interconnection between the LIG and the Cu electrode (Figure 1a‐vii). At this time, Ag‐NPs ink application is performed on a hot plate at 140°C to demonstrate the proposed local interconnections through rapid volatilization of the solvent in ink. By the 3D reservoir structure, these characteristics make precise local interconnection formation with high reproducibility and yield. This approach selectively confines Ag NPs to the electrode reservoir to form local electrical pathways while significantly improving the controllability and reliability of the overall process.

Another advantage of the proposed interconnection scheme is its excellent mechanical durability. The proposed reservoir electrode enables a stable interconnection between the dissimilar materials of the metal electrode and the LIG, even when the device is bent in a flexible situation. In a LIG‐metal interconnection based on Ag‐NPs ink using a conventional method, when the device is bent, stress is concentrated in the brittle LIG‐Ag region, which can easily break. This stimulation can eventually lead to the failure of the device. In contrast, the interconnection developed in this study has thick, flexible, yet durable metal electrodes that serve as structural buffers to protect the interconnection and continuously maintain stable electrical contact even under bending conditions (Figure 1a). As a result, the method proposed in this study presents a technological solution that can effectively overcome the trade‐off between electrical performance and miniaturization and the limitations of low mechanical durability that existing LIG‐based devices have faced.

To prove the concept, we demonstrated the proposed interconnection structure for miniaturization while securing excellent electrical and mechanical bonding properties between LIG and Cu electrodes through various device implementations (Figure 1b–d). First, we successfully fabricated a millimeter‐scale LIG electronic device using the proposed interconnection with a diameter of 1 mm (Figure 1b). Based on excellent flexibility and mechanical stability, a highly reliable and flexible bending sensor could also be implemented (Figure 1c). In addition, it was confirmed that the present method has excellent process compatibility with various materials and can be applied to small three‐electrode electrochemical sensors composed of complex LIG structures and silver/silver chloride (Ag/AgCl) electrode combinations (Figure 1d). In this way, the proposed interconnection technology can be utilized as a core basic technology supporting the practicality and scalability of LIG‐based electronic devices in various application fields. Details on the fabrication and application of each sensor are described in the Experimental Section/Methods Section.

**FIGURE 2:**
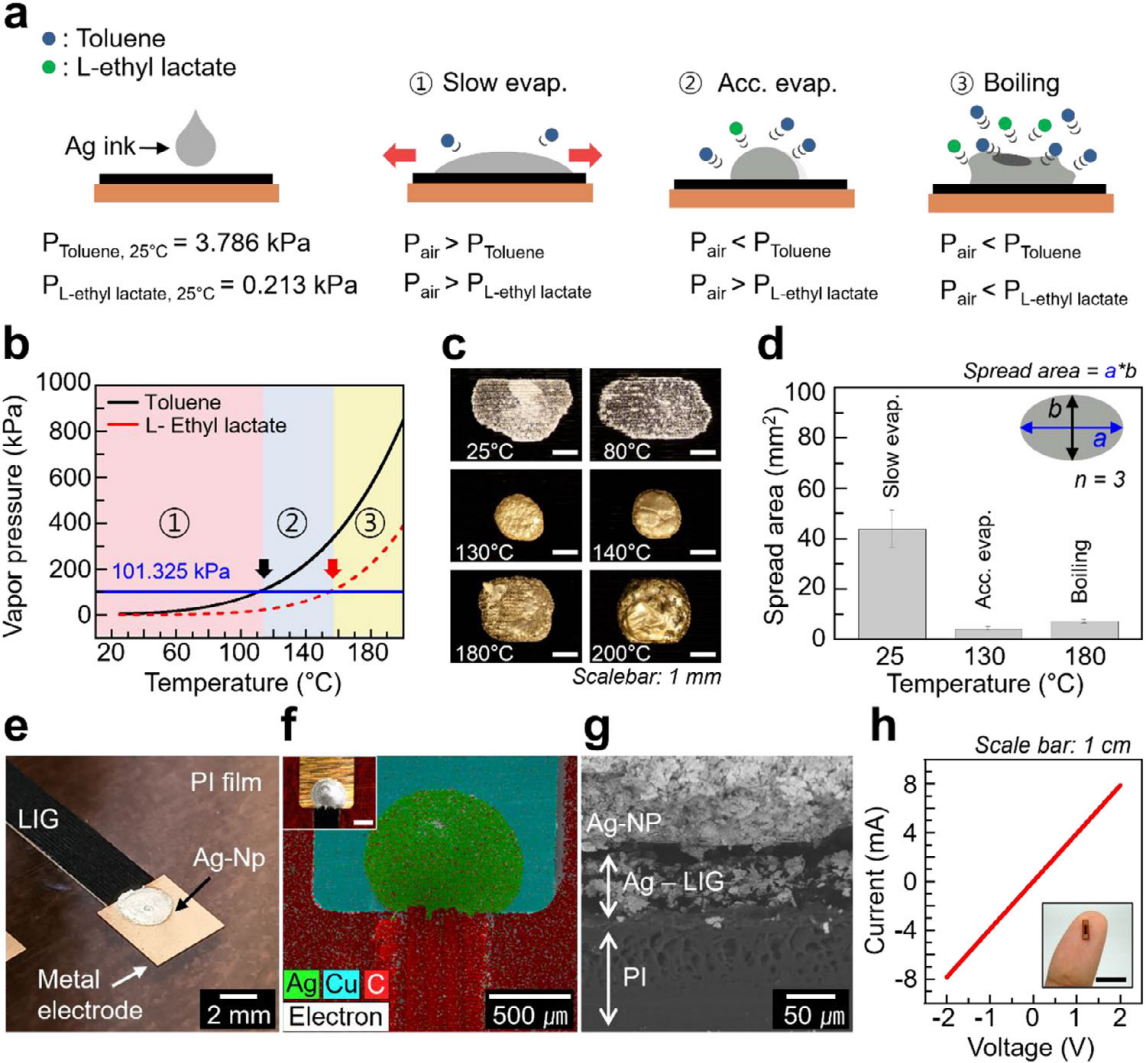
Theoretical and experimental investigation of localized Ag ink dispensing. (a) Schematic illustration of the evaporation process of the silver ink solvents (toluene and L‐ethyl lactate) under various temperature conditions. (b) Relationship between vapor pressure and temperature for toluene and L‐ethyl lactate based on the Clausius‐Clapeyron equation. (c) Optical microscopy (OM) images of Ag ink dispensed at different temperatures, and (d) comparison of the surface coverage of 5 µL Ag ink under varying thermal environments. (e) OM image of the device showing Ag‐NPs soldering interconnection. (f) EDS mapping image of the micrometer‐scale interconnection (reservoir diameter: 1 mm). (g) Cross‐sectional SEM image of the interconnection, illustrating Ag‐NPs interwoven with the structure. (h) I‐V characteristics of the fabricated micrometer‐scale device.

The most critical factor in successfully implementing the proposed concept is reliably forming the Ag‐NPs soldering in a localized area inside the 3D metallic reservoir electrode. To realize the localized soldering, we developed a quantitative method to selectively accelerate solvent evaporation by considering the Ag‐NPs ink components' vapor pressure characteristics. The commercial ink used in this study consists of 40–50 wt.% Ag, 20–30 wt.% toluene, 15–25 wt.% L‐ethyl lactate, and 5–15 wt.% acrylic resin. Typically, the ink diffuses in a solution state immediately after dispensing, during which the solvents (toluene and L‐ethyl lactate) evaporate, and the binder (acrylic resin) forms a mechanically strong conductive interconnection. That is, when Ag‐NPs ink is dropped on LIG in general usage, the solution slowly evaporates according to the volatilization characteristics of each component, and it hardens while spreading over a wide area (Figure 2a‐①).

To minimize the ink spreading and realize the localized soldering, we focused on the correlation between temperature and vapor pressure of the solvent. The vapor pressure required for a substance to change from a liquid to a gaseous state is determined as $\ln\frac{P_{1}}{P_{2}}=\frac{\Delta H_{\mathrm{vap}}}{R}\;\left(\frac{1}{T_{2}}-\frac{1}{T_{1}}\right)$ according to the Clausius‐Clapeyron equation [44, 45]. Here, P_1_ and P_2_ are the vapor pressures of substance 1 and substance 2 at absolute temperatures T_1_ and T_2_, respectively, R is the gas constant, and ΔH_vap_ is the enthalpy of vaporization. Based on Equation 1, the vapor pressure of toluene and L‐ethyl lactate, the main components of the Ag‐NPs ink solvent, can be predicted as a function of temperature.

To experimentally verify the effect of this phenomenon, the substrate on which LIG was formed was placed on a hot plate. Then, Ag‐NPs ink was dispensed in 5 µL using a micropipette from a height of 2 mm under various temperature conditions. First, soldering was performed with the Ag‐NPs ink widely spread at temperatures below the boiling points of both toluene and L‐ethyl lactate, between 25°C and 80°C. The solution strongly spreads in the direction of the laser scan, and it could be confirmed that Ag NPs were cured over an area of several mm or more overall (Figure 2a‐①,c). On the other hand, in the experiments conducted at 130°C and 140°C, which are higher than the boiling point of toluene, the Ag NPs spread in a more confined manner. As shown in Figure 2b, toluene has a vapor pressure of ∼3.8 kPa at room temperature and exceeds atmospheric pressure above its boiling point of 110.6°C. At 140°C, toluene's vapor pressure is much higher than that of L‐ethyl lactate, leading to its preferential and rapid evaporation. This behavior is confirmed by thermogravimetric analysis (TGA) and derivative thermogravimetry (DTG), which show distinct weight‐loss peaks corresponding to the boiling points of toluene and L‐ethyl lactate (Figure S4). Also, it is worth noting that the TGA/DTG results indicate that a fraction of the solvent remains even at temperatures approaching 140°C, suggesting that the ink system retains partial fluidic characteristics during the drying process. Under these conditions, preferential evaporation of toluene can generate a surface tension gradient within the ink due to compositional changes, potentially inducing Marangoni‐driven flow [46, 47]. At the same time, the relative enrichment of L‐ethyl lactate, which has a higher intrinsic surface tension, contributes to an increase in the average surface tension of the remaining solvent mixture. Concurrently, solvent loss leads to viscosity enhancement, while progressive resin solidification restricts nanoparticle mobility. These coupled effects, including evaporation‐induced Marangoni flow, gradual increase in average surface tension, viscosity rise, and binder‐assisted stabilization, collectively limit excessive spreading of the ink and enable the formation of localized and structurally stable interconnections during drying (Figure 2a‐②). This behavior is supported by molecular dynamics simulations showing that solvent evaporation occurs on very short timescales at the molecular level, which helps prevent uncontrolled ink spreading [48]. Temperature‐dependent solvent spreading results are provided in Figure S5.

**FIGURE 3:**
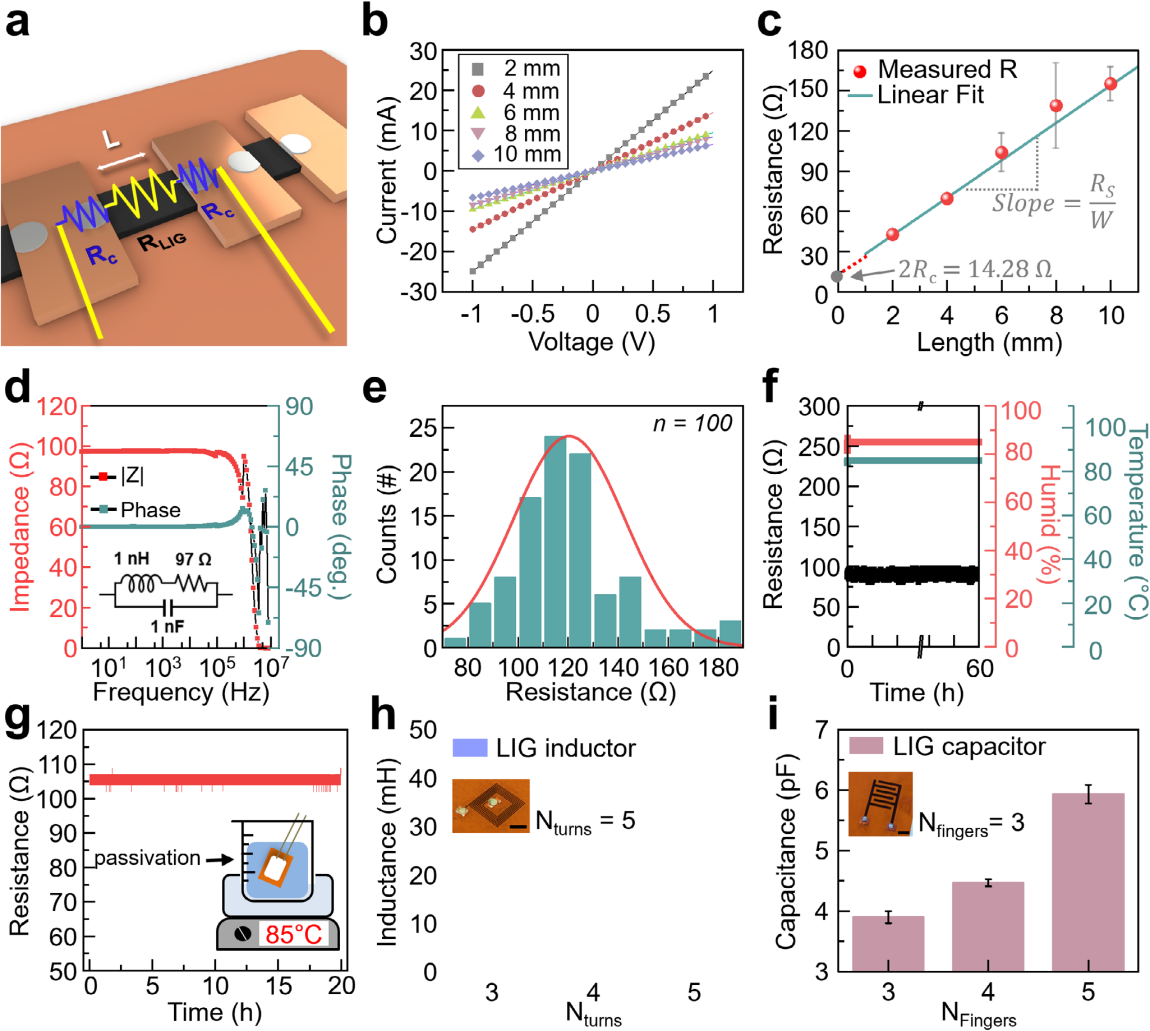
Electrical characterization of the proposed interconnection. (a) Electrical schematic illustration of the interconnection for the TLM method. (b) *I‐V* curve of the device with varying LIG lengths. (c) The result of the Transmission Line Method (TLM) analysis. (d) Impedance‐frequency (Z‐Freq) characteristics of the interconnection (inset: equivalent circuit diagram). (e) Histogram of the resistance distribution in the fabricated devices (*n* = 100). (f) Stability assessment using an 85/85 accelerated degradation test (85°C, 85% RH). (g) Resistance variation of the passivated device during 20‐h immersion in 85°C water. (h) LIG planar square coil and comparison (inset: scale bar 10 mm). (i) Capacitance measurement of the LIG capacitor (inset: scale bar 5 mm).

However, this evaporation‐driven localization mechanism is effective only within a limited temperature range. When the processing temperature exceeds 180°C, which is above the boiling points of both toluene and L‐ethyl lactate, non‐uniform soldering is observed. At such elevated temperatures, the additional vaporization of L‐ethyl lactate induces rapid boiling of the entire solvent system (red dashed line in Figure 2b). Consequently, vapor bubbles form in locally high‐energy regions, generating voids within the interconnection (Figure 2a‐③). This bubble formation promotes random redistribution of Ag‐NPs, ultimately resulting in a rough and irregularly hardened surface.

To quantitatively analyze the localized soldering area, we extracted the cured Ag NPs area. For the sake of simplicity, the product of the width and height is simplified as the spread area (inset in Figure 2d). The result shows a noticeably different spreading area depending on the temperature at which the dispensing was performed. Both toluene and L‐ethyl lactate show a wide‐spreading characteristic of several tens of mm^2^ (43.81 ± 7.53 mm^2^, *n* = 3) at temperatures below the boiling point. However, in the proposed method, a minimal spreading result of less than 5 mm^2^ with low variance can be confirmed at 130°C (4.08 ± 0.66 mm^2^), above the boiling point of toluene alone. The spreading characteristic increases again at 180°C (7.02 ± 0.77 mm^2^), above all liquids' boiling points. Moreover, to evaluate the applicability range of the proposed method, the behavior according to the volume of ink applied was experimentally analyzed. When the optimized temperature condition (140°C) was applied to the different volumes of Ag ink, it was confirmed that the spreading area tended to increase with low deviation as the ink volume increased (Figure S6). This result suggests that the proposed interconnection formation method has high process reproducibility and scalability for electrode structures of various sizes and is also effective for fabricating micrometer‐scale interconnections of multiple sizes.

Maintaining the metallic state of Ag after high‑temperature (140°C) processing is critical for stable electrical conductivity. EDS analysis confirmed excellent oxidation resistance at 140°C (Figure S7 and Table S1). This is consistent with previous findings that Ag exhibits a negligible oxidation rate at the optimized temperature [49] and can achieve stable metallic interconnections without oxide interference in this temperature range [50]. These results confirm that Ag remained metallic and the interconnection is thermally stable, leading to the observed retention of low electrical resistance.

The versatility of the proposed method was further demonstrated by fabricating a miniature metal‐LIG‐metal resistor device with the successful formation of local interconnections in the form of Ag‐NPs entrapped inside the Cu electrode reservoir (Figure 2e). The electrodes of the device were further patterned using a laser patterning process, allowing electrode diameters down to 100 µm, and at this scale, the electrodes and interconnections remain well defined, with the Ag‐NPs ink confined to the intended regions without lateral spreading into the adjacent LIG network (Figure 2f and Figure S8). Electrical characterization confirms stable device operation even at the reduced electrode dimensions. Electrical characterization confirms stable operation at reduced electrode dimensions. As the process is based on a CO_2_ laser (λ = 10.6 µm), the feature size is fundamentally limited by wavelength‐scale effects. Interconnections were formed down to ∼50 µm (Figure S9), whereas continuous LIG formation was suppressed at ≤25 µm due to diffraction‐induced energy attenuation [51, 52, 53], indicating a practical miniaturization limit of ∼50 µm under the present conditions. These results demonstrate that the proposed method operates near the practical resolution capability of CO_2_ laser processing.

The proposed interconnection method structurally confirms that the Ag‐NPs closely interconnect the LIG‐Cu electrodes. The cross‐sectional scanning electron microscope (SEM) image of the interconnection shows that the Ag‐NPs are tightly covering the LIG and interconnected to the Cu electrode (Figure 2g). In addition, we confirmed the infiltration of Ag‑NPs into the three‐dimensional porous structure of LIG at the LIG–Ag‑NPs interface. To further illustrate this, additional FE‐SEM imaging and EDS elemental mapping of the interface were performed, and the results exhibit the structural interlocking and consistent Ag distribution within the LIG pores (Figures S10 and S11). Finally, examining the *I‐V* characteristics of the LIG bar‐patterned sample with 1 mm diameter interconnections (inset of Figure 2h), the linear *I‐V* characteristics observed in the range of −2–2 V experimentally confirm that the developed method connects LIG and metal electrode with an ideal Ohmic contact (Figure 2h).

Based on the proposed concept enabling the localized heterojunctions between 3D graphene and metal electrodes, we conducted an evaluation of the resulting electrical characteristics. For the evaluation, various Cu‐LIG‐Cu devices were fabricated using the proposed interconnection approach, with length variations of the LIG segment (length = 2, 4, 6, 8, and 10 mm) (Figure S12). Then, on the devices, the transmission line method (TLM) was applied to evaluate the contact resistance (R_C_) of the developed interconnection method (Figure 3a). All measured *I–V* curves showed linear characteristics, indicating that stable Ohmic contact characteristics were formed in all devices (Figure 3b). Afterward, we calculated the contact resistance and the surface resistance by fitting linear regression analysis, respectively (Figure 3c). The interconnection exhibited a contact resistance as low as 7.14 Ω, which is significantly lower than that obtained from conventional interconnection methods widely adopted in previous studies, including Cu tape–only contacts, conductive paste combined with Cu tape, and interconnections fabricated via direct writing of nozzle‐dispensed conductive ink using a PCB printer (V‐One, Voltera, Waterloo, Ontario, Canada). Although the printed conductive ink electrode exhibited a similarly low contact resistance, its mechanical stability was insufficient due to the absence of a binder within the ink matrix, resulting in premature delamination during mechanical durability testing (Figure S13 and Table S2). This notable contact resistance reduction of the proposed interconnection is attributed to the conformal wetting of the 3D graphene surface by liquid‐phase Ag‐NPs and their infiltration into the porous graphene network, which collectively enhances the electrical coupling. Additionally, to provide a more rigorous and up‐to‐date comparison, we compiled recent interconnection approaches, including laser‐assisted metallization [9] and transfer‐printed interconnects [54, 55], in Table S3. Based on the reported quantitative metrics, the proposed method demonstrates competitive miniaturization capability, lower electrical contact resistance, and more robust mechanical durability relative to these recently reported approaches.

To assess the AC electrical performance of the proposed interconnection strategy, we fabricated bar‐shaped LIG resistor elements featuring a 2 mm‐diameter reservoir and a 6 mm‐long LIG channel. Impedance and phase responses across a range of frequencies were characterized via Bode plot analysis (Figure 3d). The LIG element exhibits stable impedance (∼97 Ω), with consistent phase characteristics up to ∼10^5^ Hz. At higher frequencies (f > 10^5^ Hz), a rapid decrease in impedance accompanied by a phase shift is observed. This behavior can be captured by a simple lumped‐element equivalent circuit comprising a series resistor (≈97 Ω) and inductor (≈1 nH), each shunted by a capacitor (≈1 nF). The high‐frequency impedance drop is attributed to parasitic capacitance and inductance associated with the two‐lead configuration, similar to that observed in commercial leaded resistors. Most LIG devices and applications, such as flexible sensors and wearable electronics, operate well below these high frequencies, so these parasitic effects are negligible. For high‐frequency LIG devices, designs minimizing parasitic capacitance and inductance, such as surface‐mount configurations, are recommended. The proposed device exhibits frequency characteristics comparable to a commercial Carbon Composition Resistor (CCR) with the same DC resistance, indicating its potential for practical applications as a stable resistor (Figure S14). To further evaluate the reproducibility of the developed interconnection, 100 units of LIG resistor elements were fabricated, and the resistance follows a normal distribution with an average resistance of 120.7 Ω (Figure 3e). The devices were fabricated across three separate batches on different days, showing no significant performance variation (Figure S15), which confirms excellent batch‐to‐batch reproducibility. It suggests a stable and consistent fabrication process, reflecting high uniformity in product quality. This high reproducibility is largely attributed to the use of reservoirs, as resistors fabricated on flat electrodes without reservoirs show lower reproducibility, indicating that selective evaporation alone is insufficient to fully confine the ink (Figure S16).

Next, we further evaluate the long‐term stability and durability of the proposed method. For the test, we first utilize accelerated life tests of the devices. The LIG elements formed interconnections using the proposed method and were stored in an environment of relative humidity of 85% and a temperature of 85°C, and the resistance change over time was tracked. As a result, it was confirmed that the resistance was stably maintained without significant change for 60 h (Figure 3f). Also, the developed interconnection can be maintained without noticeable degradation of its performance even when passivated with an additional layer (Figure S17). The passivated device with a commercial marine epoxy (Marine Epoxy, Loctite) maintains its properties for over 20 h, even in water at a high temperature of 85°C (Figure 3g). From these results, we can conclude that the proposed method retains its performance under harsh conditions when combined with a protective layer, indicating its strong potential for application in high‐performance LIG‐based electronic devices in environments such as in vivo or underwater. Additionally, the proposed method can also be utilized to demonstrate various miniaturized passive elements with high reliability. Inductors and capacitors with multiple turns and electrode overlap structures were manufactured, and their inductance and capacitance were measured. As a result, the fabricated inductance was 5–40 mH, and the fabricated capacitance was 3.0–6.5 pF (Figure 3h,I). Through this, it was experimentally proven that the proposed interconnection method can be effectively and stably applied to the design and implementation of various LIG‐based passive components without degrading the characteristics of the components.

**FIGURE 4:**
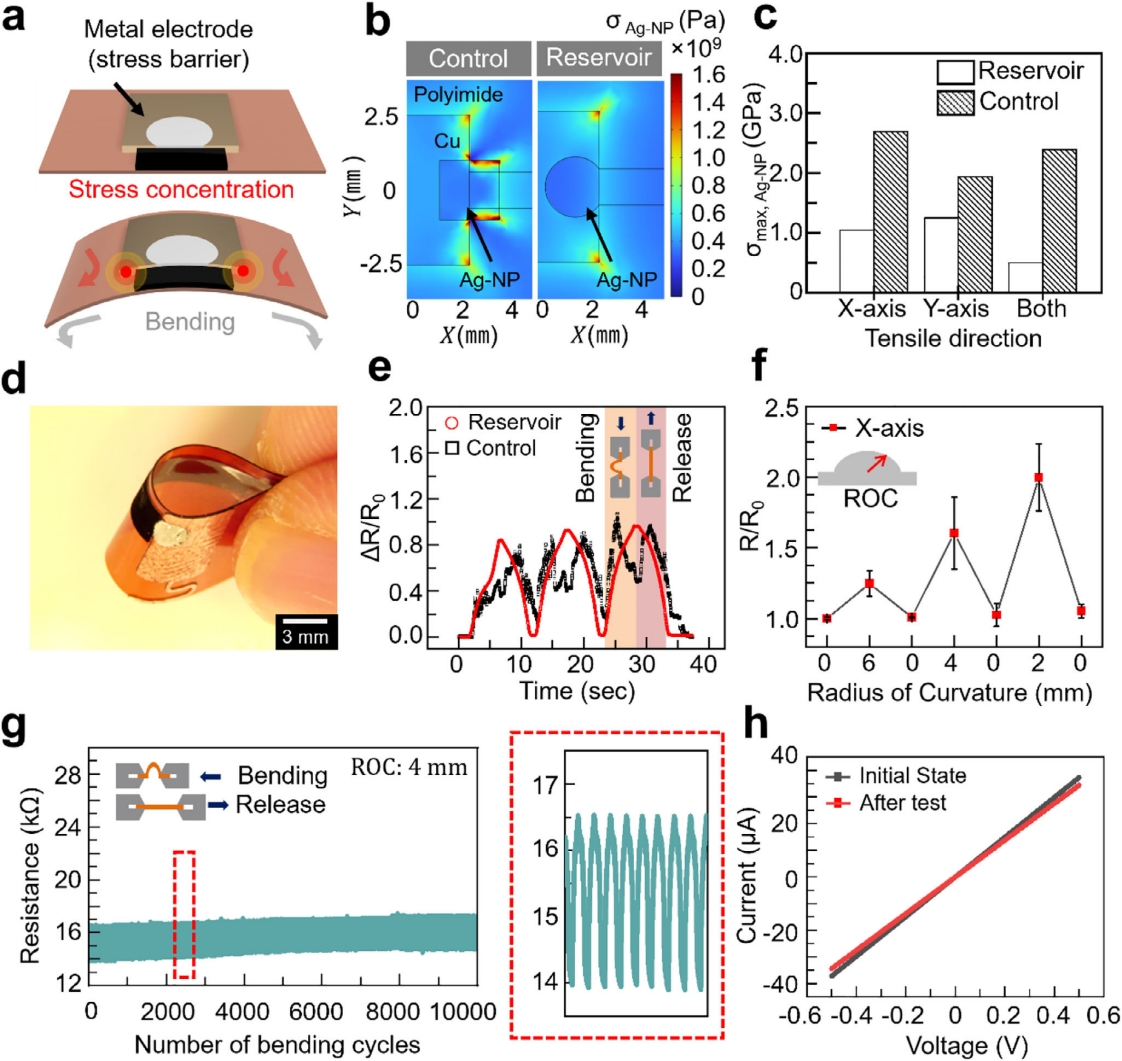
Mechanical characterization of the proposed interconnection. (a) Schematic illustration of the FEM simulation setup. (b) FEM simulation results showing von Mises stress distribution (color bar indicates stress level). (c) Comparison of maximum von Mises stress in Ag‐NP soldering for the typical (Control) and proposed structures. (d) The device's bending image. (e) Resistance change rate after three bending cycles of LIG devices fabricated using typical and damage‐suppressing (Reservoir) methods. (f) Resistance variation of the LIG bending sensor under different bending radii. (g) Bending cycle test results without passivation. (h) I‐V characteristics of the device before and after 10000 bending cycles.

The developed interconnection locally stabilizes the electrical contact between the LIG and the metal through the concave reservoir structure inside the metal electrode while effectively dispersing the mechanical stress in a flexible environment, thereby improving both the electrical properties and mechanical durability (Figure 4a). To verify the effect of the proposed structure on the stress attenuation effect applied to the interconnection, a finite element analysis (FEM) simulation was performed using COMSOL Multiphysics (Figure S18). For simulation, the structure of 5 mm by 5 mm Cu electrodes with an Ag interconnection in a reservoir structure (diameter of 2 mm) on a flexible PI film substrate. Then, the von Mises stress induced in the interconnection was calculated under biaxial tensile deformation, with ± 8% strain applied along both the x‐ and y‐axis directions (Figure 4b), which corresponds to a strain level more than twice the maximum surface strain estimated during the bending situation (see Supplementary Methods for details). To demonstrate the durability enhancement of the developed interconnection, a typical method of connecting the electrode and LIG with Ag paste, rather than a reservoir structure, was also set as a control group, and a simulation of the induced stress for substrate deformation was conducted (Control in Figure 4b). Figure 4b visually shows the induced stress results for substrate deformation calculated by the finite element method.

In both cases, high stress is induced at the edges of the rigid Cu electrode due to the stretching of the substrate. However, the stress induced in the interconnection clearly shows different results from the typical and proposed methods. For the typical interconnection, it can be visually confirmed that significantly high stress is induced in the Ag interconnection portion protruding outward due to the elongation of the substrate, resulting in mechanical fracture. In contrast, the proposed interconnection method noticeably reduced the stress induced by the interconnection located inside the reservoir electrode. It can be understood that it is not transmitted even when the substrate is stretched. When quantitatively comparing the maximum stress in the interconnection, it was found that under biaxial tensile deformation with ± 8% strain applied along both the x‐ and y‐axes, the proposed interconnection design reduced the maximum von Mises stress by approximately 79% compared to the control structure. These results demonstrate that the proposed interconnection method effectively protects the interconnection under flexible or stretching conditions, enabling more stable and reliable operation of electrical devices (Figure 4c).

To experimentally verify the improved mechanical durability, the resistance changes of the LIG resistor element manufactured by the developed method were experimentally confirmed according to a bending test. First, we manually bent the flexible metal–LIG–metal device fabricated using the proposed method and observed no visible fractures, with the device maintaining its original structural integrity (Figure 4d). To assess mechanical stability in a more controlled and quantitative manner, we conducted a bending test using an automated push–pull gauge system (force gauge (M5‐2) and stand (ESM303), Mark‐10, USA). The devices fabricated with the proposed interconnection were subjected to three repeated bending cycles (bending radius of curvature = 4 mm). For the comparison, the same tests were also conducted on the devices fabricated with the typical interconnection (Figure S19). As a result, in the case of the typical interconnection method elements, the electrical signal according to bending is very unstable, indicating the device failure (Black square line in Figure 4e). However, in the case of the proposed method, a continuous and reversible resistance change is shown according to bending (Red circle in Figure 4e), which can be considered as the result of the developed interconnection having excellent durability against mechanical deformation, unlike the typical method. Even after three actual tests, observations showed that cracks occurred in the interconnections of the typical devices, but no noticeable fractures were observed in the proposed interconnections (Figure S20).

The interfacial shear and peel strength of the Ag‐NPs/LIG/Cu interconnection was evaluated and found to be consistent with mechanically robust interfaces reported for flexible and wearable electronics [56, 57, 58] (Figure S21). Finite element analysis further indicated that, under 8% biaxial tensile strain, the average out‐of‐plane stress applied to the interconnection (∼2.09 kPa) remains well below the experimentally measured normal adhesion strength (∼11.2 kPa), supporting the mechanical reliability of the heterojunction under practical deformation conditions (Figure S22). In addition, a supplementary tape test confirmed that the sample maintained both structural and electrical integrity over more than 100 attachment–detachment cycles, with no observable delamination or performance degradation. Together, these results demonstrate that the interconnection exhibits stable mechanical durability suitable for flexible and wearable applications [57, 59].

We further tested the device stability fabricated by the proposed method concerning the radius of curvature of the device's bending. It was confirmed that the device's resistance tends to change depending on the substrate radius of curvature (Figure 4f). Specifically, the developed interconnection shows a resistance change when the degree of bending increases from ROC = 6 mm to 2 mm. Notably, it shows reversible characteristics that return to the original resistance value even when returning to the flat state after bending. It confirms that the developed interconnection method maintains stable characteristics even at a high strain level of ROC = 2 mm. In addition, even when the device was repeatedly bent 10000 times without separate passivation through repetitive bending at a ROC of approximately 4 mm, the developed resistor showed stable and reversible resistance change characteristics (Figure 4g,h). While this result demonstrates the intrinsic mechanical robustness of the device, the incorporation of additional passivation layers is recommended to further mitigate baseline drift and enhance long‐term stability under more demanding or extended bending conditions.

Accordingly, although the device exhibits intrinsic mechanical robustness, the incorporation of additional passivation layers is recommended to further mitigate baseline drift and enhance long‐term stability under repeated bending.

**FIGURE 5:**
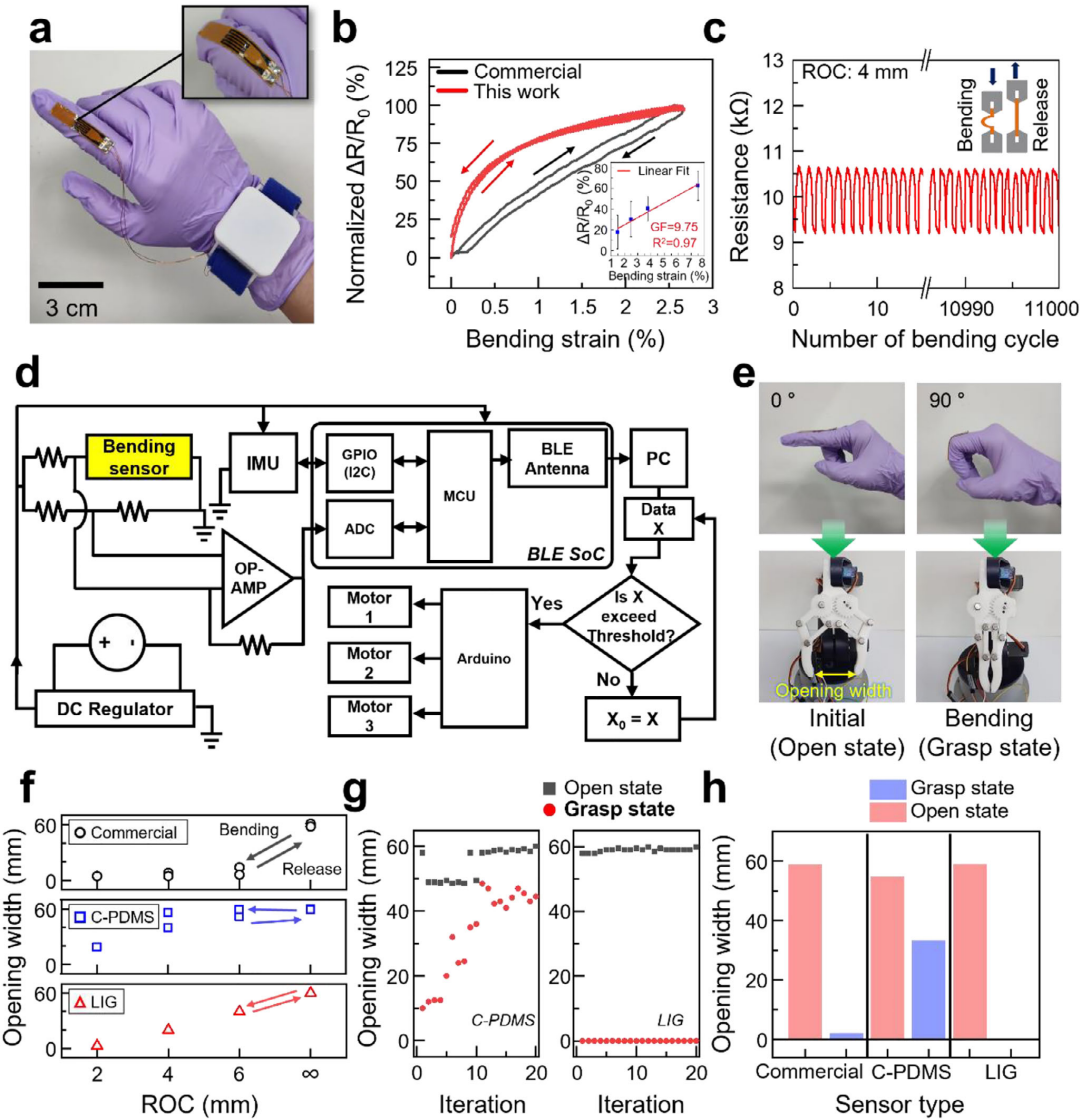
LIG‐based physical bending sensor integrated with the proposed interconnection. (a) Image of the LIG bending embedded in a watch‐type wearable device. (b) Performance comparison between the proposed LIG bending sensor and a commercial flex sensor (Short Flex Sensor: ID P1070). (c) Durability test results of the LIG bending sensor under 11000 bending cycles. (d) Block diagram of the system used for robotic arm control. (e) The gripper image according to the finger's bending angle. (f) Gripper opening width of the robotic arm in response to a single bending–releasing cycle, based on ROC signals obtained from a commercial sensor, a C‐PDMS sensor, and the LIG sensor. (g) Gripper opening width during 20 repeated bending–releasing cycles using the C‐PDMS and LIG sensors. (h) Quantitative comparison of the gripper opening width performance.

The developed interconnection has reliable electrical contact characteristics and exhibits high suitability for mechanical durability and miniaturization. These characteristics enable the realization of LIG electrical devices for emerging electrical applications, such as point‐of‐care diagnostics and human‐robot interfaces. First, we fabricated a wearable LIG‐based bending sensor designed as a strain gauge attached to a finger to precisely detect bending along a finger's length (Figure 5a). For a stable acquisition of electrical signals, the contact between the LIG of the sensor and the Cu electrode was implemented using the interconnection at the proximal phalanx position. Additionally, the sensor was finally packaged through PDMS spin coating for more stable bending durability, and the specific fabrication process and structure are described in detail in Figure S23 and Experimental Section. Under conditions of 3% bending strain, the sensor exhibited a stable resistance increase, resulting from the piezoresistive effect, where the morphological deformation induced within the microstructure of the LIG due to bending is reflected as an increase in electrical resistance. In strain‐sensing applications, hysteresis is a critical parameter because it directly affects signal accuracy and repeatability during cyclic mechanical motion. The developed bending sensor exhibited negligible hysteresis under repeated bending, indicating highly reproducible signal behavior. In contrast, a commercial flex sensor (Short Flex Sensor: ID P1070) under the same conditions exhibited a hysteretic resistance change (Figure 5b). The miniature and mechanically robust LIG interconnection also contributed to highly stable and repeatable sensing behavior with negligible hysteresis, which is particularly advantageous for reliable signal acquisition in dynamic robotic applications (Figure S24 and Table S4). Conventionally, the sensor utilizes conductive polymers for the sensing material. Still, these viscoelastic materials and a thick substrate with high stiffness exhibit significant hysteresis phenomena due to their high viscosity and flow characteristics, leading to decreased sensor reliability. Details of comparing quantitative hysteresis are shown in Figure S25. In addition, the developed sensor showed a gauge factor of 9.75 within 8% bending strain (inset Figure 5b), which can also be interpreted as obtaining enhanced piezoresistive characteristics of LIG due to low contact resistance at the contact portion. The interconnection experimentally demonstrated excellent durability and repeatability by exhibiting only negligible resistance changes even after more than 11,000 repeated bending tests (ROC = 4 mm) (Figure 5c). Consequently, the device demonstrates reliable and robust operation with negligible hysteresis and extremely high repeatability, making it suitable for practical implementation in flexible electronics, especially in wearable sensor applications.

The sensor performance was evaluated by bending the finger from 0° to 90° in 10° increments, during which the resistance exhibited a clear and consistent change with increasing bending strain, demonstrating reliable responsiveness (Movie S1). These compact, high‐reliability wearable bending sensors are well‐suited for human‐robot interface applications, where accurate and repeatable feedback is essential.

Importantly, the developed sensor exhibits negligible hysteresis, ensuring that repeated bending to the same angle produces virtually identical resistance values, a key advantage for precise and reversible control. To achieve precise control, the sensor output must remain stable even under subtle and repetitive body movements. We implemented a multimodal motion sensing system by integrating the wearable bending sensor with a 9‐axis IMU capable of detecting dynamic movements, and demonstrated its effectiveness in real‐time robot control (Figure 5d). Based on the proposed system algorithm, the gripper's opening width was continuously modulated in response to resistance changes induced by finger bending (Figure 5e). Notably, owing to the negligible hysteresis and high signal fidelity of the developed LIG‐based sensor, the system enabled reliable and repeatable gripper control that could not be achieved with conventional flexible sensing technologies, which typically suffer from signal drift and hysteresis under repetitive motion (Figure 5f). In contrast, a bending sensor fabricated with the same geometry and dimensions as the LIG sensor failed to provide stable real‐time feedback, resulting in irregular and non‐uniform variations in the gripper opening width during operation (Figure 5g,h, and Movie S2). A control algorithm was developed to allow the gripper to grasp objects based on finger posture: when the finger is fully extended, the gripper opens, and when bent to 90°, the gripper closes (Figure 5e and Figure S26). The *x*, *y*, *z* axis angle data can be simultaneously extracted from an integrated IMU, and this motion data is wirelessly transmitted to a PC via a wrist—worn watch‐type device, enabling real‐time control of a 3‐axis robotic arm through a custom algorithm implemented on an Arduino‐based platform. The system includes signal processing for discriminating between different motions of the elbow flexion and extension with the obtained angle data from the IMU via processing (Figure S27). The effectiveness of the interface was validated through successful object grasping tasks (Movie S3). As demonstrated in Figure 5g, the system successfully performed complex tasks such as object relocation and grasping in real time, validating the sensor's potential for integration into more sophisticated human‐robot interface applications. This result experimentally proves that the developed technology can be effectively utilized in precise motion detection and control applications, such as actual robot control.

**FIGURE 6:**
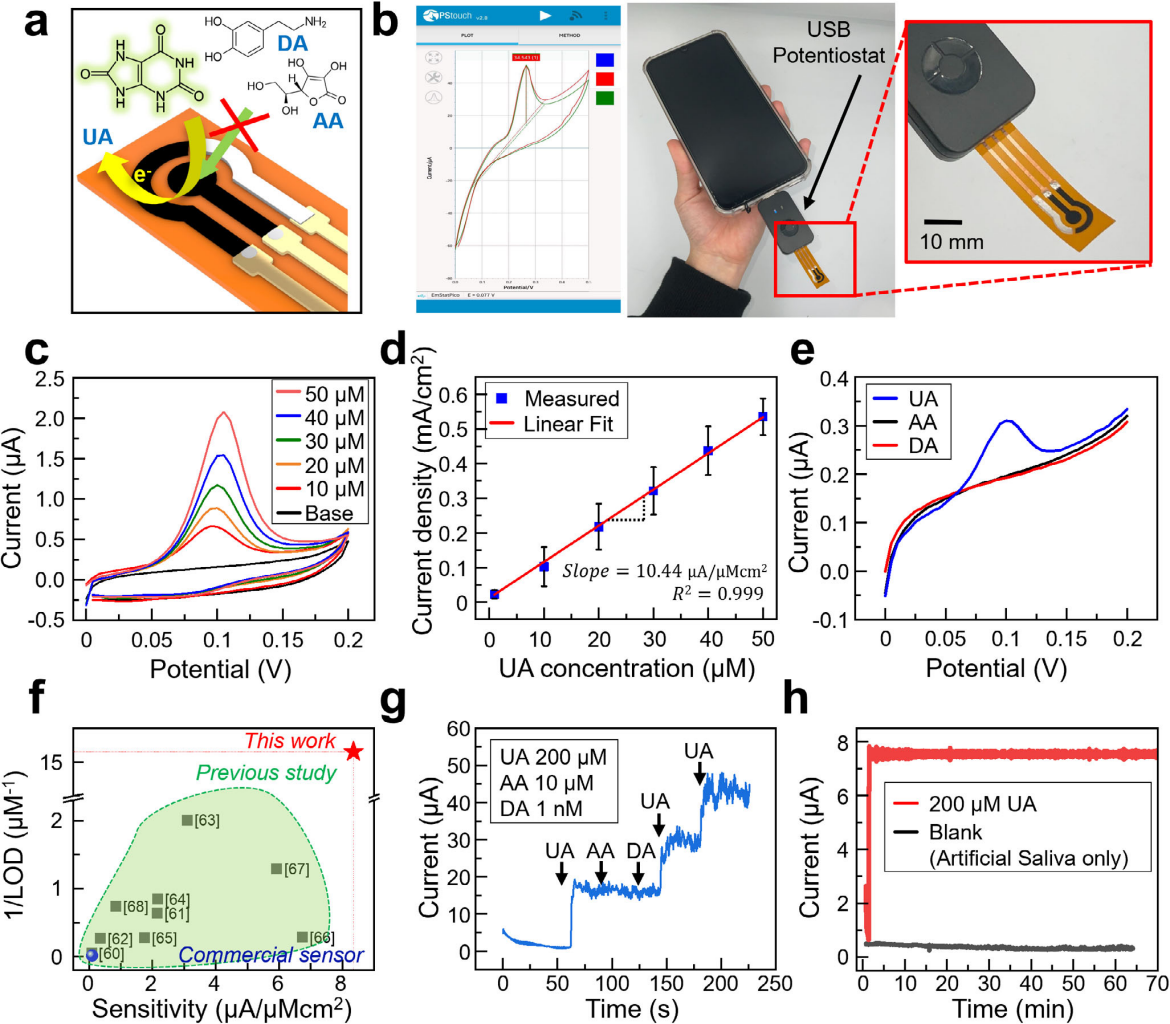
Uric Acid‐sensing LIG electrochemical sensor with the proposed LIG Interconnection. (a) Fabricated a three‐electrode system‐based uric acid sensing electrochemical LIG sensor. (b) Photographs showing LIG measurement results and sensor integration with a portable USB potentiostat. (c) Cyclic voltammetry (C‐V) measurements at uric acid concentrations ranging from 0 to 50 µm. (d) Current density response of uric acid concentration based on differential pulse voltammetry (DPV). (e) Cyclic voltammetry response when the sensor is exposed to ascorbic acid (AA), dopamine (DA), and uric acid (UA). (f) Comparison of sensitivity and inverse limit of detection (1/LOD) with previous uric acid sensor studies [60, 61, 62, 63, 64, 65, 66, 67, 68]. (g) Amperometric responses and (h) Chronoamperometric responses recorded over 1 h in the absence and presence of UA.

The interconnection method proposed in this study was also applied to implement a compact portable three‐electrode‐based electrochemical sensor. According to previous studies, LIG electrodes exhibit excellent material properties that enable the selective detection of uric acid (UA), an inflammation biomarker, by suppressing interference from various substances (Figure 6a). Although LIG‐based biosensors offer superior selectivity and high sensitivity, they have faced challenges in achieving reproducible performance and device miniaturization, limiting their applicability to point‐of‐care (POC) diagnostics. However, the interconnection technology developed in this study overcomes these limitations by enabling robust electrical connections and compact device integration, thereby enhancing the practical utility of LIG‐based electrochemical sensors in POC applications. The sensor manufactured in such a small size can be mounted on a portable smartphone potentiostat, Sensit smart (PalmSens, Netherlands), to instantly measure changes in electrochemical signals according to uric acid concentration via a smartphone (Figure 6b). Cyclic voltammetry (CV, 0‐0.2 V vs. Ag/AgCl, scan rate: 10 mV s^−1^) was performed to evaluate the electrochemical performance of the three‐electrode sensor. The result shows that no distinct redox reaction was observed in the pH 7.4 Phosphate Buffer (PB) solution without UA. Still, as UA was added, a specific oxidation peak appeared at around 0.1 V (vs. Ag/AgCl), and it was confirmed that the corresponding peak current continuously increased as the UA concentration increased (Figure 6c). Differential pulse voltammetry (DPV) analysis was performed for a more quantitative and precise sensitivity evaluation according to UA concentration (Figure S28). As a result, the DPV current density increased linearly with the UA concentration in the physiological concentration range of 10–50 µm (Figure 6d). The sensitivity of the representative three biosensors was measured to be 10.44 µAµM^−1^cm^−2^. The baseline current was 127.57 µA, and the noise level was 2.23 µA. Based on these values, the limit of detection (LOD) was 0.066 µm. Also, we confirmed that the present sensor can selectively detect UA through the difference in oxidation potential with major interfering substances such as ascorbic acid (AA) and dopamine (DA) in addition to UA, which is an essential result in high selectivity even in various in vivo interference environments (Figure 6e and Figure S29).

Finally, we compared the sensitivity and LOD of our sensor, which is the main parameter of the electrochemical sensor performance, with that of other LIG‐based UA sensors (Figure 6f, detailed information of the control sensors is in Table S5. The comparison results are interpreted as the result of the proposed interconnection method stably transmitting electrical signals without signal loss or degradation of interconnection characteristics between LIG and metal electrodes. To assess statistical significance and reproducibility, a total of 30 biosensors fabricated under the same conditions at different time points were evaluated. All 30 devices were included in the analysis without excluding any outliers. The sensitivity distribution was confirmed to follow a normal (Gaussian) behavior (Shapiro–Wilk test: W = 0.963, p = 0.368), indicating that the observed variation is consistent with expected experimental fluctuations. The devices exhibited an average sensitivity of 8.35 ± 2.37 µAµM^−1^cm^−2^ (Figure S30), with the variability arises primarily from minor process differences in manual fabrication steps. While these results demonstrate reproducibility under laboratory conditions, the observed relative standard deviation (28%) reflects the limitations of small‐scale manual fabrication. Achieving tighter performance distributions suitable for industrial applications would require systematic optimization of electrode uniformity, dispensing precision, curing conditions, and material homogeneity. Nonetheless, the current analysis provides a statistically rigorous validation that the proposed LIG–metal interconnection method can be implemented reliably at the laboratory scale, supporting its feasibility for proof‐of‐concept demonstration.

In addition, when repeated measurements were performed 10 times in 10 µm UA solutions for the same sensor, consistent oxidation peak currents were maintained within a few µA range for all samples, verifying significant repeatability (Figure S30). These findings suggest that the proposed interconnection method contributes to improved device performance and enhanced reliability and structural stability. We consider this a noteworthy and previously unreported advancement, demonstrating the high applicability of the interconnection method at the LIG device integration level within this research domain.

To further evaluate the applicability of the proposed platform under physiologically relevant conditions, the developed sensor was tested in artificial saliva containing representative salivary interferents. Dopamine (∼1 nM) and ascorbic acid (∼10 µm) were introduced at concentrations within the reported physiological ranges in human saliva [69, 70], and UA was examined within its typical salivary concentration range (about tens to hundreds of µm) [71, 72]. As shown in Figure 6g, amperometric measurements exhibited a distinct and immediate current response upon UA addition, whereas negligible signal changes were observed for dopamine and ascorbic acid, confirming selective UA detection in a saliva‐like matrix despite its increased ionic strength and compositional complexity [73, 74]. Detailed DPV analyses, showing systematic increases in current with UA concentration, are provided in Figure S31.

The operational stability was further assessed by 1 h chronoamperometric measurements under continuous bias (Figure 6h). The sensor maintained a stable baseline in artificial saliva without UA and displayed a sustained current response upon UA introduction without noticeable signal drift. Although the overall current magnitude was attenuated compared with phosphate buffer measurements, likely due to matrix‐induced effects, the device preserved stable and selective electrochemical performance, highlighting the mechanical robustness and electrical reliability of the LIG–metal interconnection platform while indicating that further interface optimization would enhance quantitative accuracy in real biofluids.

## 3 Conclusion

In this study, we developed a method for forming electrically and mechanically stable and miniaturized LIG‐metal interconnections using Ag‐NPs ink. The technology successfully performed local applications by controlling the evaporation characteristics of the solvent in the Ag‐NPs ink, and its distinguishing feature is that it exhibits high mechanical stability and reproducibility compared to the existing interconnections. The contact resistance is 7.14 Ω, which is in the few‐ohm range, and stable current changes were confirmed according to repeated bending. The developed interconnection was applied to the LIG bending sensor for robot control, and it was confirmed that it maintains the interconnection without signal distortion even after 10,000 cycles of repeated bending and relaxation with low hysteresis. Thus, the characteristic evaluation results obtained in this study demonstrate the functional capabilities and potential applications of LIG. Moreover, by adjusting ink composition and curing conditions, the proposed interconnection method can be extended to temperature‐sensitive and stretchable substrates, as long as three‐dimensional reservoir electrodes can be realized on the substrate and laser‐induced graphene can be generated or integrated. This compatibility further broadens the applicability of the proposed approach. It is expected that LIG can serve as a core material for next‐generation electronic devices with miniaturization and high performance.

## 4 Experimental Section/Methods

### 4.1 Fabrication of LIG Device for Characterization

Cu electrodes (∼10 µm thick) were formed on a 100 µm‐thick PI film via a transfer process using a PDMS adhesive layer before LIG patterning. Comparable ohmic contact was observed for thicker electrodes (∼50 µm), indicating that interconnection performance is largely independent of electrode thickness (Figure S32). LIG was then directly fabricated on the PI substrate under ambient conditions using a 10.6 µm wavelength CO_2_ laser (VLS2.30, Universal Laser Systems, USA) in raster mode, with fixed parameters: 9 W power, 31.75 mm s^−1^ scan speed, and 500 pulses per inch (PPI). During the process, LIG was patterned along the laser path, effectively filling the reservoir structure between the two electrodes. The laser was scanned laterally across the gap between the electrodes. Subsequently, Ag‐NPs ink was deposited into the structure using a micropipette while maintaining the substrate on a 140°C hot plate. All fabrication steps were carried out under atmospheric conditions.

### 4.2 Fabrication and Characterization of LIG Bending Sensor

A small‐sized LIG bending sensor was fabricated on a 12 × 40 mm piece of PI film that had been laser‐cut after being mounted on double‐sided tape. It was finally fabricated by batch passivation via PDMS spin‐coating (Figure S22). The developed bending sensor operates as a variable resistor, transmitting amplified signals to the ADC of the MCU (ISP 1807) through a Wheatstone bridge and an operational amplifier (OP‐AMP) without a separate filtering circuit. The sensor's mechanical operation characteristics were evaluated regarding resistance change and bending using an automated displacement control system and a commercial source measurement unit (B2902A, Keysight, USA).

### 4.3 Fabrication and Characterization of LIG Electrochemical Sensor for UA Detection

For the electrochemical sensor fabrication, we patterned both the working electrode (WE, area 8.34 mm^2^) and the counter electrode (CE) using LIG and a liquid Ag/AgCl screen‐printed as the reference electrode (RE) (Figure S33).

### 4.4 Control Algorithm and Robot Implementation

A wireless robot control system was implemented by integrating sensor signal processing with real‐time motor actuation. Three‐axis orientation data were acquired from an inertial measurement unit (IMU) at 5 Hz. Before the operation, gyroscope offset and magnetic field calibrations were performed to ensure accurate orientation measurements. Raw angle data were processed using coordinate transformations, with yaw calculated as Z′ = ((angleZ+180) % 360)−180 and pitch computed as X′ = (angleX−angleY). The processed angles were mapped to motor‐specific pulse‐width modulation (PWM) ranges and transmitted wirelessly via Bluetooth Low Energy (BLE) to an Arduino microcontroller, which controlled the motors. The total system latency was measured to be below 200 ms, enabling real‐time robotic response and precise control.

## Conflicts of Interest

The authors declare no conflicts of interest.

## Supporting information




**Supporting File 1**: advs76273‐sup‐0001‐SuppMat.docx.

**Supporting File 2**: advs76273‐sup‐0002‐MovieS1.mp4.

**Supporting File 3**: advs76273‐sup‐0003‐MovieS2.mp4.

**Supporting File 4**: advs76273‐sup‐0004‐MovieS3.mp4.

## Data Availability

Data supporting this study are available from the corresponding author upon reasonable request.

## References

[1] H. G. Shin , W. K. Chung , and K. Kim , “Soft and Flexible Robot Skin Actuator Using Multilayer 3D Pneumatic Network,” Nature Communications 16 (2025): 5575, 10.1038/s41467-025-60496-9. PMC12216154 40595478

[2] C. Wu , H. Liu , S. Lin , J. Lam , N. Xi , and Y. Chen , “Shape Morphing of Soft Robotics by Pneumatic Torsion Strip Braiding,” Nature Communications 16 (2025): 3787, 10.1038/s41467-025-59051-3. PMC12015459 40263355

[3] D. Corzo , G. Blázquez , and D. Baran , “Flexible Electronics: Status, Challenges and Opportunities,” Frontiers in Electronics 1 (2020): 594003, 10.3389/felec.2020.594003.

[4] C. Shang , Q. Xu , N. Liang , et al., “Multi‐Parameter E‐Skin Based on Biomimetic Mechanoreceptors and Stress Field Sensing,” npj Flexible Electronics 7 (2023): 19, 10.1038/s41528-023-00252-5.

[5] Q. Wang , S. Cai , G. Yao , et al., “Stretchable Wireless Optoelectronic Synergistic Patches for Effective Wound Healing,” npj Flexible Electronics 8 (2024): 64, 10.1038/s41528-024-00351-x.

[6] Y. Jiang , A. A. Trotsyuk , S. Niu , et al., “Wireless, Closed‐loop, Smart Bandage With Integrated Sensors and Stimulators for Advanced Wound Care and Accelerated Healing,” Nature Biotechnology 41 (2023): 652–662, 10.1038/s41587-022-01528-3. 36424488

[7] R. R. Nair , M. Nita‐Lazar , V. R. Badescu , et al., “Metallization of Leaf‐derived Lignocellulose Scaffolds for High‐performance Flexible Electronics and Oligodynamic Disinfection,” npj Flexible Electronics 8 (2024): 66, 10.1038/s41528-024-00353-9.

[8] S. Zhang , A. Chhetry , M. A. Zahed , et al., “On‐Skin Ultrathin and Stretchable Multifunctional Sensor for Smart Healthcare Wearables,” npj Flexible Electronics 6 (2022): 11, 10.1038/s41528-022-00140-4.

[9] X. Huang , Y. Liu , J. Zhou , et al., “Garment Embedded Sweat‐activated Batteries in Wearable Electronics for Continuous Sweat Monitoring,” npj Flexible Electronics 6 (2022): 10, 10.1038/s41528-022-00144-0.

[10] N. Gozzi , L. Chee , I. Odermatt , et al., “Wearable Non‐invasive Neuroprosthesis for Targeted Sensory Restoration in Neuropathy,” Nature Communications 15 (2024): 10840, 10.1038/s41467-024-55152-7. PMC11686223 39738088

[11] E. H. Kim , S. H. Cho , J. H. Lee , et al., “Organic Light Emitting Board for Dynamic Interactive Display,” Nature Communications 8 (2017): 14964, 10.1038/ncomms14964. PMC5399280 28406151

[12] W. Hong , C. Chen , X. Yao , et al., “Polydimethylsiloxane‐based Conductive Nanocomposites With High Stretchability, Biomimetic Structure for Ultra‐high Linearity Strain Sensor and Intelligent Temperature Monitoring,” Composite Structures 372 (2025): 119571, 10.1016/j.compstruct.2025.119571.

[13] Y. Zhao , S. Mu , B. Hu , et al., “Polyvinyl Alcohol/Silk Fibroin/Graphene Hydrogel‐based Flexible Sensor With Self‐healing, Ultra‐stretchable, and Eco‐friendly Properties for Advanced Wearable Electronics and Human‐Machine Interaction Applications,” Sensors and Actuators B: Chemical 445 (2025): 138566, 10.1016/j.snb.2025.138566.

[14] W. Hong , X. Guo , T. Zhang , et al., “Dual Bionic‐Inspired Stretchable Strain Sensor Based on Graphene/Multi‐Walled Carbon Nanotubes/Polymer Composites for Electronic Skin,” Composites Part A: Applied Science and Manufacturing (2024): 108043, 10.1016/j.compositesa.2024.108043.

[15] W. Hong , X. Guo , T. Zhang , et al., “Flexible Strain Sensor Based on Nickel Microparticles/Carbon Black Microspheres/Polydimethylsiloxane Conductive Composites for Human Motion Detection,” ACS Applied Materials & Interfaces 16 (2024): 32702–32712, 10.1021/acsami.4c04830. 38870327

[16] S. Mukherjee , A. Mukherjee , Z. Bytesnikova , et al., “2D Graphene‐Based Advanced Nanoarchitectonics for Electrochemical Biosensors: Applications in Cancer Biomarker Detection,” Biosensors and Bioelectronics 250 (2024): 116050, 10.1016/j.bios.2024.116050. 38301543

[17] K. S. Novoselov , A. K. Geim , S. V. Morozov , et al., “Electric Field Effect in Atomically Thin Carbon Films,” Science 306 (2004): 666–669, 10.1126/science.1102896. 15499015

[18] S. Bae , H. Kim , Y. Lee , et al., “Roll‐to‐Roll Production of 30‐inch Graphene Films for Transparent Electrodes,” Nature Nanotechnology 5 (2010): 574–578, 10.1038/nnano.2010.132. 20562870

[19] J. N. Coleman , M. Lotya , A. O'Neill , et al., “Two‐dimensional Nanosheets Produced by Liquid Exfoliation of Layered Materials,” Science 331 (2011): 568–571, 10.1126/science.1194975. 21292974

[20] W. S. Hummers and R. E. Offeman , “Preparation of Graphitic Oxide,” Journal of the American Chemical Society 80 (1958): 1339, 10.1021/ja01539a017.

[21] J. Lin , Z. Peng , Y. Liu , et al., “Laser‐Induced Porous Graphene Films From Commercial Polymers,” Nature Communications 5 (2014): 5714, 10.1038/ncomms6714. PMC4264682 25493446

[22] F. Han , S. Chen , F. Wang , et al., “High‐Conductivity, Self‐Healing, and Adhesive Ionic Hydrogels for Health Monitoring and Human‐Machine Interactions Under Extreme Cold Conditions,” Advanced Science 12 (2025): 2412726, 10.1002/advs.202412726. 39874215 PMC12021042

[23] S. Zhuo , Z. Wu , C. Williams , C. Sundaresan , and S. K. Ameri , “In‐Ear Electronics With Mechanical Adaptability for Physiological Sensing,” Advanced Healthcare Materials 14 (2025): 2404296, 10.1002/adhm.202404296. 39663718 PMC11773109

[24] S. Chen , J. Qi , S. Fan , Z. Qiao , J. C. Yeo , and C. T. Lim , “Flexible Wearable Sensors for Cardiovascular Health Monitoring,” Advanced Healthcare Materials 10 (2022): 2100116, 10.1002/adhm.202100116. 33960133

[25] F. Han , X. Xie , T. Wang , et al., “Wearable Hydrogel‐Based Epidermal Sensor With Thermal Compatibility and Long Term Stability for Smart Colorimetric Multi‐Signals Monitoring,” Advanced Healthcare Materials 12 (2023): 2201730, 10.1002/adhm.202201730. 36259562

[26] Z. Zhang , Y. Xu , Z. Zhang , et al., “Strain‐Sensitive and Strain‐Insensitive Flexible Electronics for Healthcare Monitoring,” Advanced Healthcare Materials 1 (2025): e03333, 10.1002/adhm.202503333. 41088774

[27] F. Han , T. Wang , G. Liu , et al., “Materials With Tunable Optical Properties for Wearable Epidermal Sensing in Health Monitoring,” Advanced Materials 34 (2022): 2109055, 10.1002/adma.202109055. 35258117

[28] J. Guo , J. Tuo , J. Sun , et al., “Stretchable Multimodal Photonic Sensor for Wearable Multiparameter Health Monitoring,” Advanced Materials 37 (2025): 2412322, 10.1002/adma.202412322. 39670687

[29] B. Dai , C. Gao , and Y. Xie , “Flexible Wearable Devices for Intelligent Health Monitoring,” VIEW 3 (2022): 20220027, 10.1002/VIW.20220027.

[30] I. Mondal , A. Zoabi , and H. Haick , “Biodegradable, Humidity‐Insensitive Mask‐Integrated E‐Nose for Sustainable and Non‐Invasive Continuous Breath Analysis,” Advanced Functional Materials 35 (2025): 2425193, 10.1002/adfm.202425193.

[31] Y. Xu , E. De la Paz , A. Paul , et al., “In‐Ear Integrated Sensor Array for the Continuous Monitoring of Brain Activity and of Lactate in Sweat,” Nature Biomedical Engineering 7 (2023): 1307–1320, 10.1038/s41551-023-01095-1. PMC10589098 37770754

[32] Y. Chen , X. Zhang , L. Wang , et al., “Low‐Power Hardware Architectures for Flexible Wearable Devices,” Device 3 (2025): 100748, 10.1016/j.device.2025.100748.

[33] X. Wu , S. Ye , C. Gao , et al., “Programmable Sewing Sensors for Preventing Neck Injuries,” Chemical Engineering Journal 525 (2025): 170044, 10.1016/j.cej.2025.170044.

[34] F. Han , P. Ge , F. Wang , et al., “Smart Contact Lenses: From Rational Design Strategies to Wearable Health Monitoring,” Chemical Engineering Journal 497 (2024): 154823, 10.1016/j.cej.2024.154823.

[35] F. Han , J. Li , P. Xiao , et al., “Wearable Smart Contact Lenses: A Critical Comparison of Three Physiological Signals Outputs for Health Monitoring,” Biosensors and Bioelectronics 257 (2024): 116284, 10.1016/j.bios.2024.116284. 38657379

[36] S. Gandla , M. Naqi , M. Lee , et al., “Highly Linear and Stable Flexible Temperature Sensors Based on Laser‐Induced Carbonization of Polyimide Substrates for Personal Mobile Monitoring,” Advanced Materials Technologies 5 (2020): 2000014, 10.1002/admt.202000014.

[37] I. Khan , N. Baig , A. Bake , et al., “Robust Electrocatalysts Decorated Three‐dimensional Laser‐induced Graphene for Selective Alkaline OER and HER,” Carbon 213 (2023): 118292, 10.1016/j.carbon.2023.118292.

[38] A. Velasco , Y. K. Ryu , A. Hamada , A. de Andrés , F. Calle , and J. Martinez , “Laser‐induced Graphene Microsupercapacitors: Structure, Quality, and Performance,” Nanomaterials 13 (2023): 788, 10.3390/nano13050788. 36903673 PMC10005378

[39] A. K. Yagati , A. Behrent , S. Beck , et al., “Laser‐Induced Graphene Interdigitated Electrodes for Label‐Free or Nanolabel‐enhanced Highly Sensitive Capacitive Aptamer‐Based Biosensors,” Biosensors and Bioelectronics 164 (2020): 112272, 10.1016/j.bios.2020.112272. 32553348

[40] Y.‐K. Yen , G.‐W. Huang , and R. Shanmugam , “Laser‐Scribing Graphene‐based Electrochemical Biosensing Devices for Simultaneous Detection of Multiple Cancer Biomarkers,” Talanta 266 (2024): 125096, 10.1016/j.talanta.2023.125096. 37651909

[41] J. Coelho , R. F. Correia , S. Silvestre , et al., “Paper‐Based Laser‐Induced Graphene for Sustainable and Flexible Microsupercapacitor Applications,” Microchimica Acta 190 (2023): 40, 10.1007/s00604-022-05610-0. PMC9803761 36585475

[42] U. C. J. R. Jaleel , S. Bhat , Y. N. Sudhakar , et al., “Laser‐Induced Graphene Electrode‐based Supercapacitors: Insight on the Influence of Aqueous Electrolytes on Its Energy Storage Potential,” Journal of Materials Science 60 (2025): 10944–10964, 10.1007/s10853-025-11069-0.

[43] S. A. Mensah , F. El‐Bab , A. M. R. Fath EL‐Bab , Y. Tominaga , and A. S. G. Khalil , “Precisely Engineered Interface of Laser‐induced Graphene and MoS_2_ Nanosheets for Enhanced Supercapacitor Electrode Performance,” Applied Surface Science 688 (2025): 162230, 10.1016/j.apsusc.2024.162230.

[44] R. Clausius , “Ueber Die Bewegende Kraft der Wärme und die Gesetze, Welche Sich Daraus für die Wärmelehre Selbst Ableiten Lassen,” Annalen der Physik 155 (1850): 368–397, 10.1002/andp.1850155030626.

[45] J. Thompson and A. Paluch , “Revisiting the Clausius/Clapeyron Equation and the Cause of Linearity,” Thermo 3 (2023): 412–423, 10.3990/thermo3030025.

[46] S. Karpitschka , F. Liebig , and H. Riegler , “Marangoni Contraction of Evaporating Sessile Droplets of Binary Mixtures,” Langmuir 33 (2017): 4682–4687, 10.1021/acs.langmuir.7b00740. 28421771 PMC5645759

[47] H. Gelderblom , C. Diddens , and A. Marin , “Evaporation‐Driven Liquid Flow in Sessile Droplets,” Soft Matter 18 (2022): 8535–8553, 10.1039/D2SM00931E. 36342336 PMC9682619

[48] S. Cheng and G. S. Grest , “Molecular Dynamics Simulations of Evaporation‐Induced Nanoparticle Assembly,” The Journal of Chemical Physics 138 (2013): 064701, 10.1063/1.4789807. 23425482

[49] E. Ide , S. Angata , A. Hirose , and K. F. Kobayashi , “Metal‐to‐metal Bonding Process Using Ag Metallo‐organic Nanoparticles,” Sensors and Actuators A: Physical 121 (2005): 527–532, 10.1016/j.sna.2005.03.022.

[50] S. J. Hong , J. W. Lim , C. H. Choi , et al., “Low‐Temperature Thermal Oxidation of Ag Thin Films,” Journal of Materials Science: Materials in Electronics 16 (2005): 315–319, 10.1007/s10854-005-0925-5.

[51] X. Ruan , R. Wang , J. Luo , Y. Yao , and T. Liu , “Experimental and Modeling Study of CO_2_ Laser Writing Induced Polyimide Carbonization Process,” Materials & Design 160 (2018): 1168–1177, 10.1016/j.matdes.2018.10.050.

[52] M. He , Y. Wang , S. Wang , and S. Luo , “Laser‐Induced Graphene Enabled 1D fiber Electronics,” Carbon 168 (2020): 308–318, 10.1016/j.carbon.2020.06.084.

[53] E. Hecht , Optics. 5th ed. (Pearson, 2017).

[54] J. Ren , D. Li , Y. Zhang , W. Yang , H.‐Y. Nie , and Y. Liu , “Laser Direct Activation of Polyimide for Selective Electroless Plating of Flexible Conductive Patterns,” ACS Applied Electronic Materials 4, no. 5 (2022): 2191–2202, 10.1021/acsaelm.1c01193.

[55] D. Song , A. Mahajan , E. B. Secor , M. C. Hersam , L. F. Francis , and C. D. Frisbie , “High‐Resolution Transfer Printing of Graphene Lines for Fully Printed, Flexible Electronics,” ACS Nano 11, no. 7 (2017): 7431–7439, 10.1021/acsnano.7b03795. 28686415

[56] Y. Lu , et al., “Stretchable Graphene–hydrogel Interfaces for Wearable and Implantable Bioelectronics,” Nature Electronics 7, (2024): 51–65, 10.1038/s41928-023-01091-y.

[57] J. Kim , A. S. Campbell , B. E.‐F. de Ávila , and J. Wang , “High‐Adhesive Flexible Electrodes and Their Manufacture: A Review,” Micromachines 12 (2021): 1505, 10.3390/mi12121505. 34945355 PMC8704330

[58] Y. Zhang , Z. Liu , Q. Liu , et al., “Enhancing the Interfacial Binding Strength Between Modular Stretchable Electronic Components,” Advanced Functional Materials 30 (2020): 2004277, 10.1002/adfm.202004277.

[59] M. Seong , H.‐H. Park , I. Hwang , and H. E. Jeong , “Strong and Reversible Adhesion of Interlocked 3D‐Microarchitectures,” Coatings 9 (2019): 48, 10.3390/coatings9010048.

[60] Y. Zhao , X. Yan , Z. Kang , et al., “Highly Sensitive Uric Acid Biosensor Based on Individual Zinc Oxide Micro/Nanowires,” Microchimica Acta 180 (2013): 759–766, 10.1007/s00604-013-0981-z.

[61] V. Nagal , S. Masrat , M. Khan , et al., “Highly Sensitive Electrochemical Non‐enzymatic Uric Acid Sensor Based on Cobalt Oxide Puffy Balls‐Like Nanostructure,” Biosensors 13 (2023): 375, 10.3390/bios13030375. 36979587 PMC10046517

[62] B. Kulyk , S. O. Pereira , A. J. S. Fernandes , E. Fortunato , F. M. Costa , and N. F. Santos , “Laser‐Induced Graphene From Paper for Non‐enzymatic Uric Acid Electrochemical Sensing in Urine,” Carbon 197 (2022): 253–263, 10.1016/j.carbon.2022.06.013.

[63] L. Zhang , L. Wang , J. Li , C. Cui , Z. Zhou , and L. Wen , “Surface Engineering of Laser‐induced Graphene Enables Long‐term Monitoring of on‐body Uric Acid and pH Simultaneously,” Nano Letters 22 (2022): 5451–5458, 10.1021/acs.nanolett.2c01500. 35731860

[64] V. Kammarchedu , D. Butler , and A. Ebrahimi , “A Machine Learning‐based Multimodal Electrochemical Analytical Device Based on eMoSx‐LIG for Multiplexed Detection of Tyrosine and Uric Acid in Sweat and Saliva,” Analytica Chimica Acta 1232 (2022): 340447, 10.1016/j.aca.2022.340447. 36257734

[65] I. Anshori , A. R. Adzkia , Uperianti , et al., “Uric Acid Electrochemical Biosensor Based on a Laser‐Induced Graphene Electrode Modified With a Honey‐Mediated Nanocomposite of Reduced Graphene oxide and Bimetallic Silver–Cobalt,” RSC Advances 15 (2025): 39431–39442, 10.1039/D5RA04596G. 41122398 PMC12536868

[66] A. Joshi and G. Slaughter , “Cost‐Effective Hierarchical Cobalt Nanostructured Laser‐Induced Graphene for Enhanced Uric Acid Detection,” Advanced Sensor Research 4 (2025): e70003, 10.1002/adsr.70003.

[67] S. Choudhury , S. Zafar , D. Deepak , A. Panghal , B. Lochab , and S. S. Roy , “A Surface Modified Laser‐Induced Graphene Based Flexible Biosensor for Multiplexed Sweat Analysis,” Journal of Materials Chemistry B 13 (2025): 274–287, 10.1039/D4TB01936A. 39535206

[68] P. Zhao , Y. Zhang , Y. Liu , D. Huo , J. Hou , and C. Hou , “Wearable Electrochemical Patch Based on Iron Nano‐Catalysts Incorporated Laser‐Induced Graphene for Sweat Metabolites Detection,” Biosensors and Bioelectronics 249 (2024): 116012, 10.1016/j.bios.2024.116012. 38232450

[69] S. Boobalan , L. Leelavathi , and S. Jayaraman , “Comparative Assessment of Salivary Dopamine and Acetylcholinesterase Levels in Smokers and Non‐Smokers,” Journal of Pioneering Medical Sciences 14, no. S01 (2025): 328–333, 10.47310/jpms202514S0141.

[70] E. Mäkilä and P. Kirveskari , “A Study of Ascorbic Acid in Human Saliva,” Archives of Oral Biology 14, no. 11 (1969): 1285–1292, 10.1016/0003-9969(69)90201-5. 5260892

[71] J. Zhao and Y. Huang , “Salivary Uric Acid as a Noninvasive Biomarker for Monitoring the Efficacy of Urate‐lowering Therapy in a Patient With Chronic Gouty Arthropathy,” Clinica Chimica Acta 450 (2015): 115–120, 10.1016/j.cca.2015.08.005. 26276048

[72] A. Jaiswal , S. Madaan , N. Acharya , S. Kumar , D. Talwar , and D. Dewani , “Salivary Uric Acid: A Noninvasive Wonder for Clinicians?,” Cureus 13, no. 11 (2021): 19649, 10.7759/cureus.19649. PMC8675576 34956769

[73] J. Liu , Y. Tang , Y. Cheng , W. Huang , and L. Xiang , “Electrochemical Biosensors Based on Saliva Electrolytes for Rapid Detection and Diagnosis,” Journal of Materials Chemistry B 11 (2023): 33–54, 10.1039/d2tb02031a. 36484271

[74] Y. Liu , K. J. Aoki , and J. Chen , “The Difference in the Effects of IR‐Drop From the Negative Capacitance of Fast Cyclic Voltammograms,” Electrochem 4, no. 4 (2023): 460–472, 10.3390/electrochem4040030.

