# Supplementary material for: Bending‐Resistant Intimate 3D Graphene–Metal Heterojunctions for Highly Sensitive and Robust Flexible Sensors
Source: Adv Sci (Weinh). 2026 Jul 2;13(52):e00080. doi: 10.1002/advs.202600080 (PMC13336879; doi:10.1002/advs.202600080)
Supplement: Supplementary file 1 [file ADVS-13-e00080-s003.docx]

Supporting Information

**Bending-Resistant Intimate 3D Graphene–Metal Heterojunctions for Highly Sensitive and Robust Flexible Sensors**

*Saeyoung Park^1†^, Yoo-Kyum Shin^1†^, Na-Kyoung Yang^1^,* Gyeong-Hwan Park^2^, Somi Lee^1^, and Min-Ho Seo^1,2^*

^1^Department of Information Convergence Engineering, College of Information and Biomedical Engineering, Pusan National University, Yangsan, Republic of KOREA

^2^School of Biomedical Convergence Engineering, College of Information and Biomedical Engineering, Pusan National University, Yangsan, Republic of KOREA

*^†^* These authors contributed equally: Saeyoung Park, Yoo-Kyum Shin

* Corresponding authors: mhseo@pusan.ac.kr (M.-H.S.)

**Figure S1. Three-dimensional surface profile of the reservoir-structured Cu electrodes fabricated via the transfer method.** The electrodes have a thickness of approximately 70 µm and a surface roughness (Sa) of 0.82 µm.

**Figure S2. XPS of the thick film electrode before and after lasing.** The binding energies of the normalized Cu2P is at 932.6 eV, and there is any peak where O1s (530 eV) and C1s (284.6 eV). Raman spectra taken before and after laser exposure show no significant changes in major peaks, suggesting no chemical changes to the thick copper electrode.

**Figure S3. Optical image of the thin metal film and thick metal film electrode after lasing.** The 100 nm deposited metal electrode damage occurred by the lasing, and undesirable LIG was formed.

**Figure S4.** Thermogravimetric analysis (TGA) and derivative thermogravimetry (DTG) of Ag-NPs ink under N₂ gas with a heating rate of 20 °C/min. The TGA curve shows the overall weight loss as a function of temperature, while the DTG curve highlights the temperature(s) corresponding to maximum decomposition rates.

**Figure S5. Optical microscopy images of the 5 µL solvent-only samples as a function of processing temperature**. The significant boiling of both solvent can make non-uniform soldering result (180 °C).

**Figure S6. Optical images and quantification of the Spread area according to different ink doses dispensed at an optimized temperature 140 °C.** It was confirmed that the spread area of the ink dispensed using a micropipette exhibited low deviation, and the area increased with the ink volume. These results indicate that the application area can be finely tuned through volume control.

**Figure S7. EDS elemental mapping images of the Ag interconnection.** (a) Cu Lα1,2, (b) C Kα1_2, (c) O Kα1, and (d) Ag Lα1. The mapping confirms that Ag is concentrated in the central region with minimal oxygen presence, while Cu and C are distributed in the peripheral areas.

**Figure S8. Results of laser-patterned miniaturized electrode fabrication and electrical characterization.** (a) Optical images of devices with different electrode sizes, (b) Corresponding magnified optical and SEM images of 200 μm and 100 μm reservoir diameter, (c) I-V characteristics for device with 100 μm diameter with and without Ag ink.

**Figure S9.** (a) Schematic illustration of the slit structure used for laser irradiation. The parameter W denotes the slit width, defined as the lateral distance between the two opposing slit edges. (b) Optical microscopy images of slit interiors after laser-induced graphene (LIG) formation with varying slit widths (W = 100, 75, 50, 25, and 10 μm). The slit was fabricated by copper-plated electrode patterning. Distinct LIG formation within the slit is clearly observed for widths down to 50 μm, whereas no evident LIG generation is detected for narrower slits (W ≤ 25 μm), indicating a critical geometric threshold for successful in-slit LIG formation.

**Figure S10. EDS elemental mapping of the Ag interconnection.** Carbon (C Kα1,2) and silver (Ag Lα1) distributions. The Ag and C signals are interpenetrated at the interfacial region, indicating intimate interfacial contact between the Ag interconnection and the LIG.

**Figure S11. FE-SEM images of the electrode–LIG–Ag interconnection.** (a) Cross-sectional image of the Cu electrode–LIG–Ag interconnection. (b, c) High-magnification FE-SEM images highlighting the intimate contact and structural interlocking between the Ag nanoparticles and the porous LIG network at the interface.

**Figure S12. TLM Test to derive contact resistance of the LIG-Cu electrode interconnection**. Fabricated Test sample has length of 10, 8, 6, 4, 2 mm.

**Figure S13. TLM test samples fabricated using conventional interconnection methods.** (a,b) Direct attachment of copper tape onto the LIG surface. (c,d) Copper tape bonded with silver paste to improve electrical stability and enhance adhesion at the LIG–Cu tape interface. (e,f) Conductive ink-based electrode with a direct-writing PCB printer with nozzle-dispensed conductive ink. (g) Optical microscopy image showing interfacial exfoliation observed during sample preparation for the bending test.

**Figure S14. Impedance measurement of the commercial Carbon Composition Resistor (CCR).** Comparison of the fabricated LIG device impedance with the CCR resistor, and it shows high similarity.

**Figure S15. Box plot comparison of device performance across three fabrication batches (n=100).**

**Figure S16. Control resistors on flat electrodes (without reservoir) under optimized thermal dispensing.** (a) I–V curves of four samples showing high variability (RSD = 60.43%, n = 4). (b) Optical images of the samples.

**Figure S17. Resistance change due to marine epoxy passivation.** Resistance of the fabricated LIG device with passivation, the resistance decreased about 10.7 %.

**Figure S18. FEM simulation model in COMSOL Multiphysics.** The simulation model was used to investigate the effect of the reservoir structure of the interconnection on max stress attenuation by comparing it with the typical method in which the interconnection is positioned at the outside of the electrode.

**Figure S19. Typical interconnection fabrication process.** The typical interconnections for the stability comparison with proposed interconnection, bar pattern of metallic electrode was set us a typical type of the LIG interconnection forming method.

**Figure S20. Proposed and Typical interconnection after 3 cycles bending.** Photographic comparison of devices employing the proposed and typical interconnections before and after three repeated bending cycles.

**Figure S21. Mechanical adhesion characterization of the Ag-NPs solder-based interconnection.** (a) Optical image of the shear test sample and schematic illustration of the shear test configuration. (b) Adhesion force as a function of lateral displacement and corresponding shear stress converted from the adhesion force, yielding shear stress values of 220.07 kPa, 340.30 kPa, and 412.63 kPa, with an average shear stress of 324.33 ± 97.27 kPa. (c) Change in electrical resistance as a function of repeated attachment–detachment cycles during the tape test, demonstrating stable electrical performance over more than 100 cycles, with the resistance remaining within 1% of the initial value.

**Figure S22. COMSOL bending simulation (3D) results showing the normal stress distribution in the Ag-NP interconnection under a total biaxial (x-axis) tensile strain of 8% applied to the substrate**. (a) Simulation model and (b) the color map of the out-of-plane (z-direction) normal stress, and the average normal stress within the Ag-NP interconnection region was calculated to be 2.09 kPa.

**Figure S23. Fabrication schematic of LIG bending sensor.**

**Figure S24. Comparison of Commercial, C-PDMS, and Fabricated LIG Sensors and Experimental Setup for Gripper Motion Detection.** (a) Photographs of the commercial sensor, C-PDMS sensor, and the fabricated LIG sensor. (b) Experimental setup and schematic illustrating the extraction of the relationship between the robotic gripper opening width and the sensor response (ROC).

**Figure S25. Hysteresis area comparing with commercial flex sensor.** Resistive bending sensors have hysteresis due to differences in restoring force depending on bending and flexure. A quantitative comparison was conducted by comparing this difference through integration, and the fabricated LIG-based bending sensor showed lower hysteresis than the commercial flex sensor.

**Figure S26. IMU-based robotic arm control results.** (a) Digital output results as a function of finger bending angle. (b) Directional control of a robotic arm using an IMU and gripper actuation according to finger bending (see **Movie S3**).

**Figure S27. Flowchart of the algorithm of robot control system and angle data processing.** Implemented algorithm to distinguish arm movements by transforming raw angle data and applying defined thresholds.

**Figure S28. Differential pulse voltammetry (DPV) was performed to precisely observe the oxidation reaction in response to varying uric acid (UA) concentration.** The oxidation peak current increased with increasing UA concentration from 0 to 50 μM.

**Figure S29. Amperometric response of the biosensor for uric acid (UA) in the presence of potential interferents.** Sequential addition of 10 µM UA, 10 µM dopamine (DA), and 10 µM ascorbic acid (AA) was performed to assess the sensor’s selectivity. The biosensor exhibited a clear response to UA while showing negligible current changes for DA and AA, confirming high selectivity. All measurements were conducted under identical conditions, and the applied potential was set to 0.7 V vs. Ag/AgCl.

**Figure S30. Evaluation of fabrication reproducibility and measurement repeatability of the UA electrochemical biosensor.** (a) Sensitivity distribution histogram (n = 30). (b) Current values from repeated sensor measurements (n = 3, at uric acid concentration = 10 µM).

**Figure S31.** Differential Pulse voltammetry (DPV) responses recorded in artificial saliva at varying UA concentrations, showing a concentration-dependent increase.

**Figure S32.** I-V curve of the resistor with proposed interconnection of 50 μm Cu electrode.

**Figure S33.** Fabrication schematic of three-electrode electrochemical LIG sensor.

**Table S1.** Chemical composition of the Ag interconnection determined by EDS analysis.

**Table S2.** Comparison of representative LIG interconnection methods reported in the literature and this work, summarizing contact resistance, bending durability, and minimum feature size. References indicate representative studies in which the corresponding interconnection methods were employed.

**Table S3.** Comparison of representative LIG interconnection methods reported in the literature and this work, summarizing contact resistance, bending durability, and minimum feature size. References indicate representative studies in which the corresponding interconnection methods were employed.

**Table S4.** Comparison of representative LIG interconnection methods reported in the literature and this work, summarizing contact resistance, bending durability, and minimum feature.

**Table S5.** Previous researches on LIG-based UA electrochemical sensors.

Supporting Methods: *FEM simulation*

**Bending-Resistant Intimate 3D Graphene–Metal Heterojunctions for Highly Sensitive and Robust Flexible Sensors**

*Saeyoung Park^1†^, Yoo-Kyum Shin^1†^, Na-Kyoung Yang^1^, Gyeong-Hwan Park^2^, Somi Lee^1^, and Min-Ho Seo^1,2^**

^1^Department of Information Convergence Engineering, Pusan National University, Yangsan, Republic of KOREA

^2^School of Biomedical Convergence Engineering, Pusan National University, Yangsan, Republic of KOREA

*^†^* These authors contributed equally: Saeyoung Park, Yoo-Kyum Shin

* Corresponding authors: mhseo@pusan.ac.kr (M.-H.S.)

**S1. Finite Element Modeling of Strain Distribution**

Finite element simulations were conducted using **COMSOL Multiphysics (v.6.0)** to evaluate the strain and stress distributions generated around the Ag nanoparticle (Ag-NP) interconnection layer embedded between the polyimide substrate and the copper electrode. The simulations were performed using the Solid Mechanics module with linear elastic material models.

**S1.1. Material Properties**

The material constants used in the finite element model are summarized in **Table S1**. All material properties were obtained from datasheets or widely accepted values in literature.

| Material | Density (kg·m⁻³) | Young’s modulus (GPa) | Poisson’s ratio | Geometry |
| --- | --- | --- | --- | --- |
| Polyimide  (substrate / LIG) | 1420 | 2.5 | 0.34 | 5 cm × 5 cm  (square film) |
| Ag-NP ink  (interconnection) | 10500 | 70 | 0.37 | Arc-shaped region  (≈90% of a 2 mm-  diameter circle) |
| Cu electrode  (rigid) | 8960 | 110 | 0.34 | 5 mm × 5 mm  (square pad) |

**Table S1. Material parameters used in the FEM simulation.**

**S1.2. Geometry and Structural Configuration**

A polyimide square substrate (5 cm × 5 cm) was modeled as the device's substrate. A reservoir-patterned copper electrode (5 mm × 5 mm) was placed at the center of the substrate. An Ag-NP interconnection region was inserted in the reservoir structure of the electrode and the underlying substrate.

This multilayer geometry reflects the actual device layout, in which strain is transferred from the outer polyimide surface through the Ag-NP interconnection to the metal electrode.

To estimate the maximum mechanical loading during operation, an upper-bound surface strain of:

$$\varepsilon_{\text{surf}}=16\%$$

was used as a conservative limit.

**S1.3. Boundary Conditions**

The mechanical boundary conditions applied to the model are summarized below.

**Displacement on substrate edges**

- Uniform in-plane displacement was applied at all four outer boundaries of the polyimide film to mimic biaxial stretching.
  - Loading directions:
    - **Biaxial strain (x + y)**
    - **Uniaxial strain along X-axis**
    - **Uniaxial strain along Y-axis**
  - Engineering strain per axis: **8%**

**Electrode constraint**

- The copper electrode region was assigned **Rigid Motion Suppression** to prevent unwanted rigid-body translation or rotation under deformation.

These settings allow the computation of strain concentration around the Ag-NP interconnection as the substrate undergoes biaxial or uniaxial stretch.

A schematic of the applied strain field is provided in **Supplementary Fig. S1**.

**S1.4. Meshing and Solver Configuration**

A physics-controlled mesh was employed with **fine** global resolution. To accurately capture localized deformation, additional mesh refinement was applied to the Ag-NP region and to the material boundaries.

A stationary study step was used with a fully coupled direct solver to obtain the equilibrium strain distribution.

**S1.5. Output Quantities**

The primary results extracted from the simulations include:

- Local von Mises stress distribution within the Ag-NP interconnection

For quantitative comparison, the **maximum von Mises stress within the Ag-NP arc-segment interconnection region** (corresponding to ~90% of a 2-mm-diameter circle) was extracted for each loading condition.

Because failure is most likely initiated at this interconnection, the peak von Mises stress serves as the primary mechanical indicator for assessing the risk of deformation-induced fracture in the device.

**S1.6. Visualization of Strain Distribution**

To analyze the deformation behavior of the device under mechanical loading, strain fields were visualized for three loading scenarios:

(1) biaxial stretching with 8% strain applied simultaneously along the x- and y-axes

(2) uniaxial stretching along the x-axis (8%)

(3) uniaxial stretching along the y-axis (8%)

For each case, both the full-field strain map of the entire polyimide substrate and a magnified view of the Ag-NP arc-segment interconnection were obtained to highlight local strain concentration. These strain visualizations are presented in **Supplementary Figs. S2–S4**.

**Supplementary Figures**

**Fig. S1. FEM model schematic for strain simulations.** Overall model consisting of a polyimide substrate (5 cm × 5 cm), a copper electrode (5 mm × 5 mm), and an Ag-NP interconnection modeled as an arc-segment region corresponding to ~90% of a 2-mm-diameter circle.

**Fig. S2. Strain distribution under biaxial stretching (8% along both x and y directions).** Full-field von Mises strain distribution across the polyimide substrate and magnified strain map around the Ag-NP arc-segment interconnection.

**Fig. S3. Strain distribution under uniaxial stretching along the x-direction (8%).** (a) Full-field strain map of the substrate under x-axis loading and magnified strain distribution at the Ag-NP interconnection.

**Fig. S4. Strain distribution under uniaxial stretching along the y-direction (8%).** (a) Full-field strain map of the substrate under y-axis loading and magnified strain distribution at the Ag-NP interconnection.
